# Evolution of Interbacterial Antagonism in Bee Gut Microbiota Reflects Host and Symbiont Diversification

**DOI:** 10.1128/mSystems.00063-21

**Published:** 2021-05-11

**Authors:** Margaret I. Steele, Nancy A. Moran

**Affiliations:** a The University of Texas at Austin, Department of Integrative Biology, Austin, Texas, USA; University of Connecticut

**Keywords:** T6SS, bumble bee, honey bee, microbiota

## Abstract

Gram-negative bacteria frequently possess type VI secretion systems (T6SSs), protein complexes that are able to inject toxic proteins into nearby cells. Many aspects of T6SS structure and function have been characterized for model species, but less is known about the evolutionary processes that shape T6SS and effector (toxin) diversity in host-associated microbial communities. The bee gut microbiota is a simple community that has codiversified with bees for >80 million years. This study investigated how complements of T6SSs and effectors within the bee microbiota changed as bacteria and their hosts diversified into isolated species. We used protein homology to survey 198 isolate genomes of 9 Gram-negative species for genes encoding T6SS structural components; Rhs toxins, which are common T6SS effectors; and VgrG proteins, which are structural components associated with specific toxins. T6SS loci were present in 5 species clusters found only in bees, namely Apibacter spp., Gilliamella spp., Frischella perrara, “*Candidatus* Schmidhempelia bombi,” and Snodgrassella alvi. The distribution of T6SS loci suggests that at least 3 were present in the microbiota of the common ancestor of social bees and that loss of these genes in some bacterial lineages was linked to both host and bacterial speciation. Isolates differed enormously in repertoires of Rhs and VgrG proteins. We found that bacterial species employ different mechanisms for toxin acquisition and diversification and that species and strains sometimes lose the T6SS entirely, likely causing shifts in competitive dynamics within these communities.

**IMPORTANCE** Antagonistic interactions between bacteria affect diversity and dynamics of host-associated communities, including gut communities that are linked to host health. In many bacterial communities, including human and honey bee gut microbiotas, antagonism is mediated by type VI secretion systems (T6SSs) that deliver lethal toxins to competing strains. In this study, we explored how T6SSs and associated toxins have evolved in the simple, host-specific gut microbiota of honey bees and bumble bees. Using comparative genomics, we explored the conservation, recombination, horizontal transfer, and loss of T6SSs and effectors during 80 million years of evolution of this bee-associated community. We find that that patterns of T6SS loss and retention are linked to differences in biology across host species, while trends in effector diversification are mostly specific to bacterial lineages.

## Abstract (precis)

Antagonistic interactions between bacteria affect diversity and dynamics of host-associated communities, including gut communities that are linked to host health. In many bacterial communities, including human and honey bee gut microbiotas, antagonism is mediated by type VI secretion systems (T6SSs) that deliver lethal toxins to competing strains.

## INTRODUCTION

Bacteria that live within host-associated communities often have mechanisms for killing potential competitors. Many Gram-negative bacteria employ type VI secretion systems (T6SSs)—protein complexes that deliver toxic proteins into the periplasm or cytoplasm of target cells—against other Gram-negative bacteria (1). T6SSs play important roles in host-associated microbial communities, including the human gut microbiota (2–4), and influence strain competition (5, 6). Secreted components of T6SSs include a needle-like tube of Hcp proteins, a trimeric VgrG spike at the end of the needle, a tip protein containing a PAAR domain, and any protein toxins (effectors) attached to these components. Nonsecreted components include a membrane complex, baseplate, and a contractile sheath that provides the force needed to expel the needle and effectors from the cell (1, 7–12). T6SSs can deliver a diverse range of effectors, which have different cellular targets, enzymatic activities, and mechanisms of associating with the T6SS (13–15). Immunity genes, usually located immediately downstream of their cognate toxin gene, encode proteins that protect the cell against the activity of each toxin. T6SS effectors and immunity genes are sometimes exchanged between bacteria through horizontal gene transfer (16, 17). Additionally, some T6SS effectors, including Rhs-family toxins and VgrG proteins, can diversify through recombination (18–20). Gain, loss, and exchange of effector and immunity genes may therefore determine which strains can coexist within hosts.

**FIG 1:**
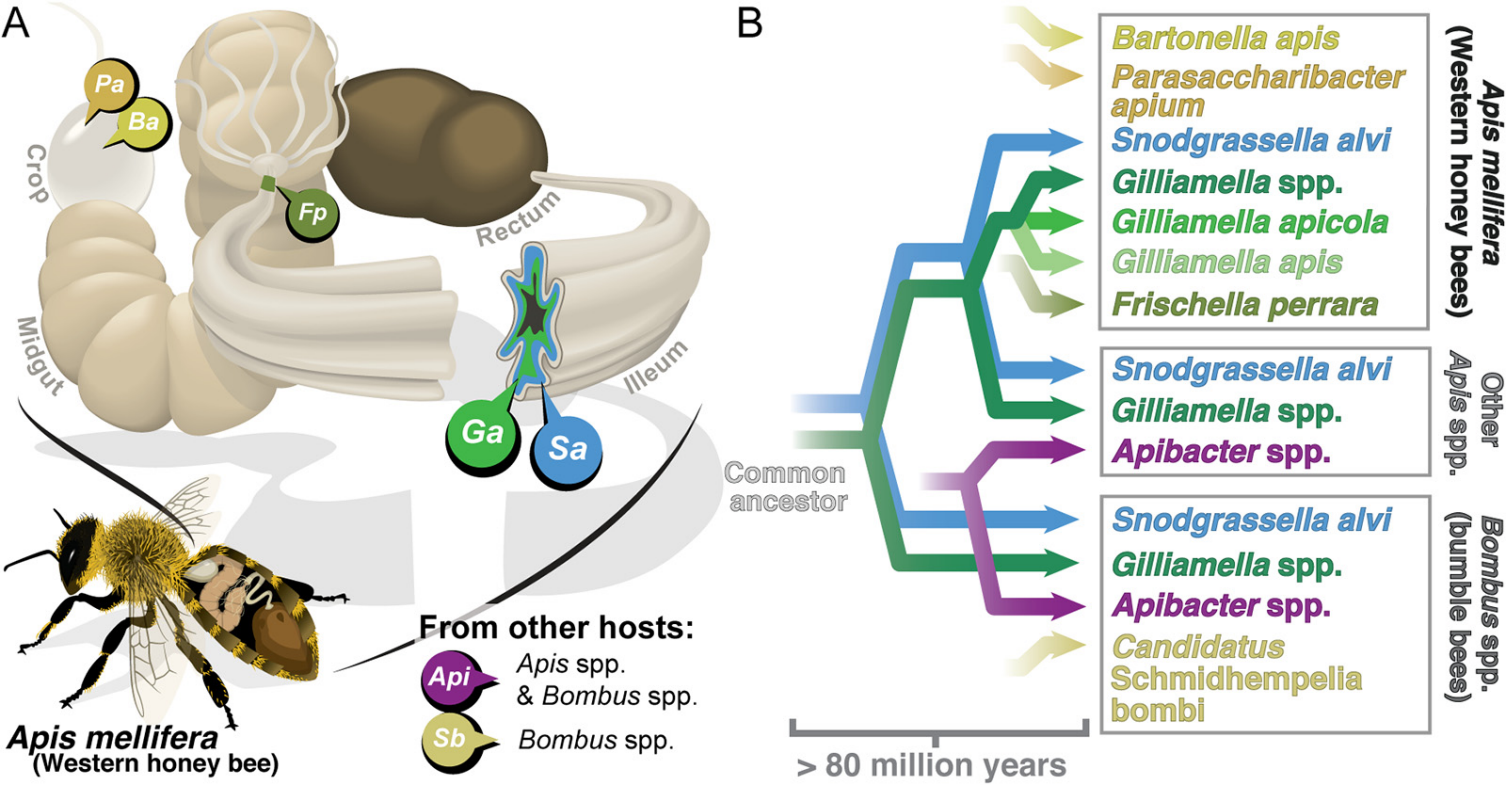
Gram-negative bacteria found in the guts of social bees. (A) Bacteria are spatially organized in the bee gut. Parasaccharibacter apium (Pa; Alphaproteobacteria) and Bartonella apis (Ba; *Alphaproteobacteria*) are found sporadically in the honey bee crop (70). Frischella perrara (Fp; *Gammaproteobacteria*) is found in many honey bees and specifically colonizes the pylorus (71, 72). *Gilliamella* spp. (Ga; *Gammaproteobacteria*) and Snodgrassella alvi (Sa; *Betaproteobacteria*) cocolonize the ileum in *Apis* and Bombus spp. (22, 43). *Apibacter* spp. (Api; Flavobacteria) are found in *Apis* and *Bombus* spp. but are uncommon in Apis mellifera (21, 32). “*Candidatus* Schmidhempelia bombi” (Sb; *Gammaproteobacteria*) is found in *Bombus* spp. (73). The location of *Apibacter* spp. and “*Candidatus* Schmidhempelia bombi” within the gut is unknown. Gram-positive bacteria associated with the bee gut are not shown. (B) Bee gut symbionts have codiversified with their hosts for more than 80 million years.

The gut microbiota of social bees provides a useful model for the evolution of host-associated communities. This simple consortium, consisting of 10 or fewer species (or closely related species clusters), is most intensively studied for the Western honey bee, Apis mellifera (Fig. 1A) (21–24). Five core bacterial lineages also form specific associations with other honey bee species (genus Apis) and bumble bees (genus Bombus). Phylogenetic reconstructions show that these 5 lineages were present in ancestral hosts and that bees and their gut bacteria have undergone long-term codiversification (21, 25–27) (Fig. 1B). We previously showed that the genomes of bee gut bacteria Snodgrassella alvi and Gilliamella apicola encode T6SSs, as well as diverse Rhs toxins, which are exchanged between strains and species and diversify through recombination (28). T6SS-mediated antagonism is likely to be important for intraspecific competition in the bee gut, where strains of the same species compete to dominate niches (26, 29), and may also play a role in interspecific competition.

The biology of bee host species likely affects the competitive dynamics within these communities. New honey bee hives are founded by swarms of hundreds to thousands of individuals, while bumble bee colonies are founded by a single female. Colonies founded by swarms are expected to have more strain diversity, as diversity is lower in individual bees than in the hive as a whole (29, 30). Furthermore, microbiota diversity in different host species correlates with both gut community size and colony size (21). This likely explains why bacterial diversity is lower in bumble bee hosts than in honey bee hosts, with a greater number of individual bumble bees dominated by a single strain of each core species (21, 27). Over millions of years of evolution, these factors could lead to dramatic shifts in T6SS function and effector diversity in the microbiotas of different bee host species.

In this study, we examined the evolution of T6SS and toxin effectors within the gut microbiotas of social bees. By analyzing multiple isolate genomes for all Gram-negative symbiont species, we compared the distribution of T6SSs across bacterial species and between gut communities restricted to different hosts. We then used a homology-based approach to look at diversification within two T6SS-secreted protein families, VgrG and Rhs, to better understand how effector evolution is shaped by recombination within and between genomes and by competitive pressures at the community level.

## RESULTS

### Bee gut symbionts retain ancestral T6SSs.

**FIG 2:**
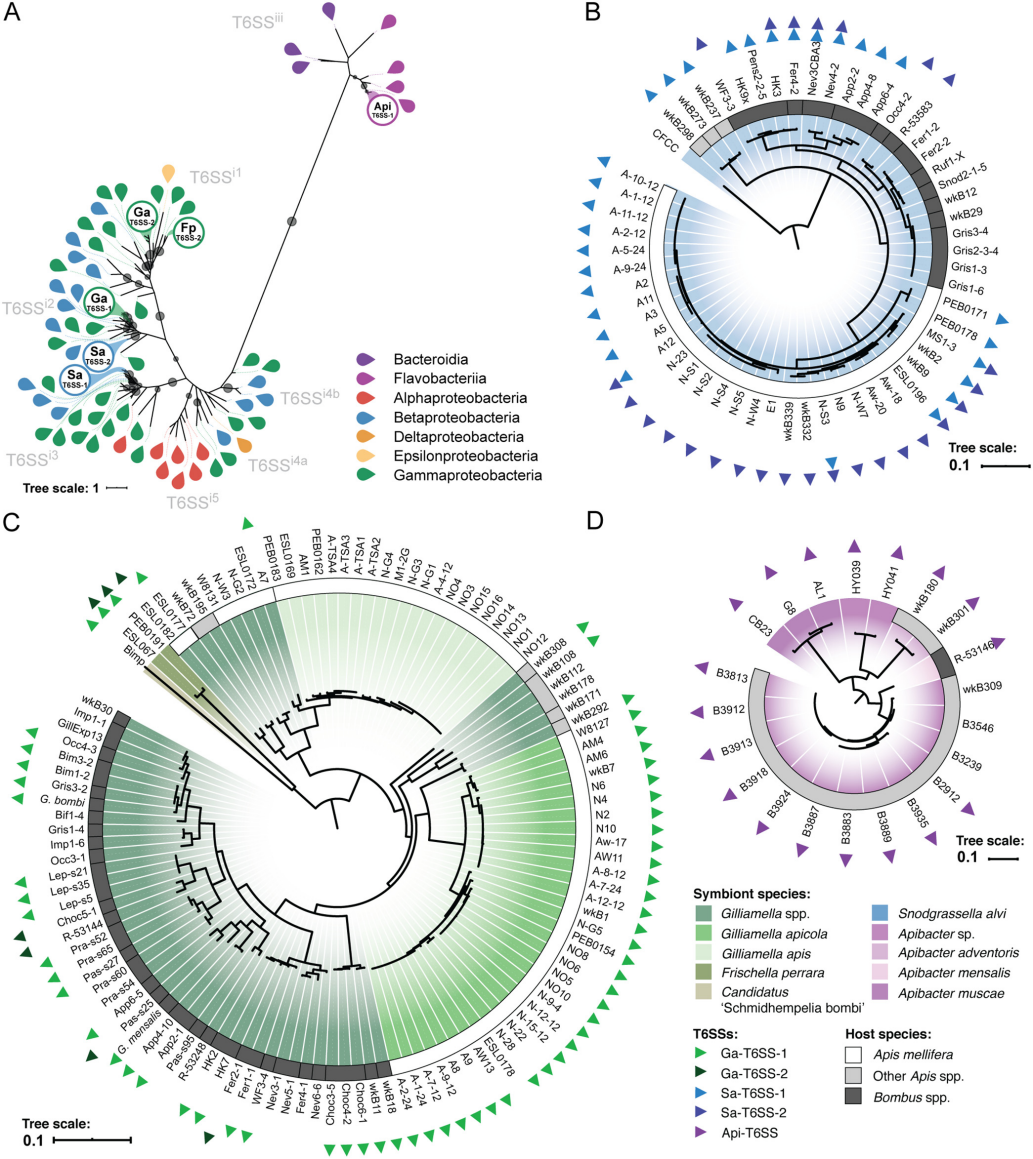
The genomes of bee gut symbionts encode multiple conserved T6SSs. (A) A maximum-likelihood phylogeny of the TssB protein, a conserved structural component of T6SSs, shows evolutionary relationships between the T6SSs of bee gut symbionts and previously described T6SS subfamilies. Leaf colors indicate the bacterial class from which each TssB sequence was extracted (further described in Table S2 in the supplemental material). Labeled circles represent TssB proteins associated with T6SS loci found in bee symbionts, as follows: Sa-T6SS-1, Sa-T6SS-2, Ga-T6SS-1, Ga-T6SS-2, Fp-T6SS-2, and Api-T6SS-1. Gray points on branches indicate >70% support with 1,000 bootstraps. (B) *S. alvi* genome phylogeny constructed from 719 single-copy gene trees. Each leaf represents a single sequenced isolate genome. The color of the ring around the phylogeny indicates the host species from which each strain was isolated: White, Apis mellifera (Western honey bees); light gray, Apis spp. (other honey bees); dark gray, *Bombus* spp. (bumble bees). Colored triangles at the outer edge of each strain name indicate that the strain genome encodes one or more T6SS loci. (C) *Gilliamella* spp. genome phylogeny, constructed from 927 single-copy gene trees, with *Frischella perrara* and “*Candidatus* Schmidhempelia bombi” as outgroups. (D) *Apibacter* spp. genome phylogeny constructed from 1,279 single-copy gene trees. Shading of phylogenies highlights different species within each genus.

In a survey of 198 isolate genomes from 9 bacterial species clusters (Fig. 1; see also Table S1 in the supplemental material), we identified complete sets of genes encoding the T6SS structural components in isolates of *Apibacter* spp., S. alvi, *Gilliamella* spp., G. apicola, Frischella perrara, and “*Candidatus* Schmidhempelia bombi,” but not in Bartonella apis, Parasaccharibacter apium, or Gilliamella apis. Maximum-likelihood phylogenies of conserved T6SS structural proteins TssB (Fig. 2A), TssC (see Fig. S1A in the supplemental material), and TssH (Fig. S1B) show that the T6SSs we identified comprise 5 distantly related clusters within previously described T6SS clades (31). Of the two T6SSs encoded by the genome of the betaproteobacterium *S. alvi* (see Fig. S2A in the supplemental material), Sa-T6SS-1 is not closely related to the T6SSs in any other bee symbiont (Fig. S2B), and the nearest relatives to Sa-T6SS-2 are T6SSs from taxa related to *S. alvi* and not associated with bees (Fig. S2C). Both Sa-T6SS-1 and Sa-T6SS-2 are found in honey bee and bumble bee symbionts and were likely present in the ancestral lineage of *S. alvi* prior to association with a bee host. Ga-T6SS-1 is found within multiple bee gut-associated Gammaproteobacteria belonging to the family Orbaceae, including *Gilliamella* spp.; “*Candidatus* Schmidhempelia bombi,” a bumble bee symbiont; and Frischella perrara, a honey bee symbiont (see Fig. S3A in the supplemental material). The Ga-T6SS-1 phylogeny is not congruent with the *Gilliamella* genome phylogeny, and it supports multiple transfers of Ga-T6SS-1 between *Gilliamella* lineages (Fig. S3B). The genomes of *F. perrara* and a few *Gilliamella* isolates encode a second T6SS locus, Ga-T6SS-2, which appears to have been transferred multiple times between *Gilliamella* spp. and *F. perrara* (Fig. S3C). The T6SS genes of *Apibacter* isolates (Api-T6SS-1) (32) are related to T6SSs found in *Apibacter* strains isolated from other animals, as well as in other members of the order Flavobacteriales (see Fig. S4 in the supplemental material). These phylogenetic analyses indicate that most of the T6SSs of modern bee gut bacteria were present in the ancestral lineages when they became symbionts. Some transfers have taken place, but these are confined to related lineages within the family *Orbaceae*.

10.1128/mSystems.00063-21.1 FIG S1 Maximum-likelihood phylogenies of (A) TssC and (B) TssH reflect the evolutionary relationships between type VI secretion systems (T6SSs) of bee gut symbionts and previously described T6SS subfamilies. Grey circles mark branches that have ≥70% support with 1,000 bootstraps. Leaf colors indicate taxonomic classes of the species from which TssC and TssH sequences were extracted. Labeled circles indicate clusters of proteins associated with the following T6SS loci found in bee symbionts: Sa-T6SS-1, Sa-T6SS-2, Ga-T6SS-1, Ga-T6SS-2, Fp-T6SS-2, and Api-T6SS-1. Download FIG S1, TIF file, 0.7 MB. Copyright © 2021 Steele and Moran. 2021 Steele and Moran. https://creativecommons.org/licenses/by/4.0/ This content is distributed under the terms of the Creative Commons Attribution 4.0 International license.

10.1128/mSystems.00063-21.2 FIG S2 Snodgrassella alvi isolates carry genes encoding two ancestral T6SSs. (A) Open reading frame (ORF) maps for Sa-T6SS-1 (*S. alvi* wkB2, GenBank accession number CP007446.1, residues 126,500 to 147,400) and Sa-T6SS-2 (accession number CP007446.1, residues 1,504,101 to 1,513,500 and 1,008,639 to 1,022,525). (B) Maximum-likelihood phylogeny for Sa-T6SS-1. The phylogeny was constructed from concatenated alignments of all proteins encoded by genes in the *S. alvi* wkB2 Sa-T6SS-1 locus, which includes 6,178 distinct alignment patterns, (C) Maximum-likelihood phylogeny for Sa-T6SS-2 based on concatenated alignments of proteins encoded within the two Sa-T6SS-2 loci in *S. alvi* wkB2, which includes 8,996 distinct alignment patterns. Both phylogenies were constructed using RAxML with the LG likelihood model for amino acid replacement and 1,000 bootstraps. Circles on branches indicate >70% support. Strain names of bee gut isolates are highlighted. Download FIG S2, TIF file, 2.4 MB. Copyright © 2021 Steele and Moran. 2021 Steele and Moran. https://creativecommons.org/licenses/by/4.0/ This content is distributed under the terms of the Creative Commons Attribution 4.0 International license.

10.1128/mSystems.00063-21.3 FIG S3 The genomes of bee-associated Orbaceae (Gammaproteobacteria) strains encode ancestral and horizontally transferred T6SSs. (A) ORF maps for Ga-T6SS-1 1 (Gilliamella apicola wkB7, GenBank accession number LZGG01000001.1, residues 2,542,845 to 2,562,752), Ga-T6SS-2 (*Gilliamella* sp. Choc5-1, accession number LZHH01000029.1, residues 1 to 25,766), and Fp-T6SS-2 (Frischella perrara PEB0191, accession number CP009056.1, residues 1,967,039 to 1,982,524 and 1,982,736 to 2,008,542). (B) Maximum-likelihood phylogeny for Ga-T6SS-1, Fp-T6SS-1, and Sb-T6SS-1 based on a concatenated alignment of homologs to proteins encoded by genes in the Ga-T6SS-1 locus of *G. apicola* wkB7. This alignment contained 7,980 distinct alignment patterns. A simplified schematic of the *Gilliamella* genome phylogeny on the right edge of the figure shows possible instances of horizontal transfer of Ga-T6SS-1 genes between distantly related *Gilliamella* strains. This phylogeny is not drawn to scale and excludes strains that lack Ga-T6SS-1; the full-genome phylogeny is shown in Fig. 2C. (C) Maximum-likelihood phylogeny for Ga-T6SS-2 and Fp-T6SS-2 based on a concatenated alignment of homologs to proteins encoded by the Ga-T6SS-2 locus of *Gilliamella* sp. Choc5-1, with 9,757 distinct alignment patterns. Both phylogenies were constructed using RAxML with the LG likelihood model for amino acid replacement and 1,000 bootstraps. Circles on branches indicate ≥70% support. Strain names of bee gut isolates are highlighted to differentiate between species clusters. Download FIG S3, TIF file, 2.5 MB. Copyright © 2021 Steele and Moran. 2021 Steele and Moran. https://creativecommons.org/licenses/by/4.0/ This content is distributed under the terms of the Creative Commons Attribution 4.0 International license.

10.1128/mSystems.00063-21.4 FIG S4 A T6SS cluster found in *Apibacter* spp. is present in isolates from bees and other animals. (A) ORF map for the Api-T6SS-1 locus in Apibacter adventoris wkB180 (GenBank accession number PSZN01000014.1, residues 1 to 15,028). (B) Maximum-likelihood phylogeny for Api-T6SS-1, constructed from a concatenated alignment of homologous proteins, containing 3,447 distinct alignment patterns. The phylogeny was constructed using RAxML with the LG likelihood model for amino acid replacement and 1,000 bootstraps Circles on branches indicate ≥70% support. Download FIG S4, TIF file, 1.8 MB. Copyright © 2021 Steele and Moran. 2021 Steele and Moran. https://creativecommons.org/licenses/by/4.0/ This content is distributed under the terms of the Creative Commons Attribution 4.0 International license.

10.1128/mSystems.00063-21.8 TABLE S1 Conserved domains found in Rhs toxins from genomes of bee gut symbionts. Download Table S1, PDF file, 0.3 MB. Copyright © 2021 Steele and Moran. 2021 Steele and Moran. https://creativecommons.org/licenses/by/4.0/ This content is distributed under the terms of the Creative Commons Attribution 4.0 International license.

10.1128/mSystems.00063-21.9 TABLE S2 Proteins used in TssB, TssC, and TssH phylogenies. Download Table S2, PDF file, 0.1 MB. Copyright © 2021 Steele and Moran. 2021 Steele and Moran. https://creativecommons.org/licenses/by/4.0/ This content is distributed under the terms of the Creative Commons Attribution 4.0 International license.

### Loss and retention of T6SSs in symbiont lineages.

We examined the retention and loss of T6SS loci in the context of the evolutionary history of bees and their gut symbionts. We previously reported, based on an analysis of genomes from 28 *S. alvi* strains collected from bees in North America and Southeast Asia, that honey bee *S. alvi* retains at least one of two vertically inherited T6SSs (Sa-T6SS-1 and Sa-T6SS-2), which are sometimes lost in *S. alvi* strains from bumble bees (28). Our updated analysis, which includes 56 *S. alvi* genomes from Europe, North America, and Asia, provides further evidence that T6SS genes are universal in *S. alvi* in honey bee guts but not in all bumble bee hosts (Fig. 2B).

Interestingly, host-specific trends in T6SS loss and retention across *Gilliamella* spp. (112 genomes) are similar to the trends seen for *S. alvi* (Fig. 2C). Among honey bee isolates, the genomes of 32 of 33 *G. apicola* strains encode a single highly conserved T6SS (Ga-T6SS-1). In contrast, *Gilliamella* spp. isolated from bumble bees differ in whether they retain T6SS genes. Ga-T6SS-1 has been lost multiple times from bumble bee *Gilliamella* strains; in some cases, it has been replaced with the distantly related Ga-T6SS-2 locus. G. apis isolates, which cooccur with G. apicola in honey bee guts, lack T6SS loci entirely. Unlike the T6SSs of other bee symbionts, Api-T6SS-1 is present in *Apibacter* spp. isolated from both bees and mammalian hosts (Fig. 2D). Api-T6SS-1 appears to have been lost in some isolates from Apis cerana. These trends suggest that differences in host biology can determine whether selective pressures favor loss or retention of T6SSs in multiple symbiont species.

### Bee gut bacterial species have distinctive repertoires of Rhs toxins.

Rhs toxins are common T6SS effectors that are easily identified by a conserved core motif (18). Diversification of *rhs* genes through recombination has been reported in multiple bacterial species (18, 19, 33), but the bee microbiota presents an opportunity to examine this process within the context of a coevolved bacterial community. Because *rhs* genes can exchange toxin domains through recombination, we performed separate analyses for core and toxin domains of Rhs proteins. Core domains are defined here as the amino acid sequence between the N terminus and a conserved DPxG motif (18) in an alignment of Rhs proteins, which includes a conserved core region and sometimes an N-terminal secretion domain. Toxin domains consist of the sequence between the DPxG motif and the C terminus. We searched the genomes of 198 bee gut isolates for proteins containing conserved Rhs core motifs, and, after filtering and clustering the sequences, we identified 208 representative C-terminal toxin domains. 64 of these toxin domains share homology with previously characterized toxin domains (Table S1). Within the bee gut isolate genomes, we found 1,528 proteins containing regions homologous to the representative toxin domains, including putative orphaned toxin domains not detected in our initial search.

**FIG 3:**
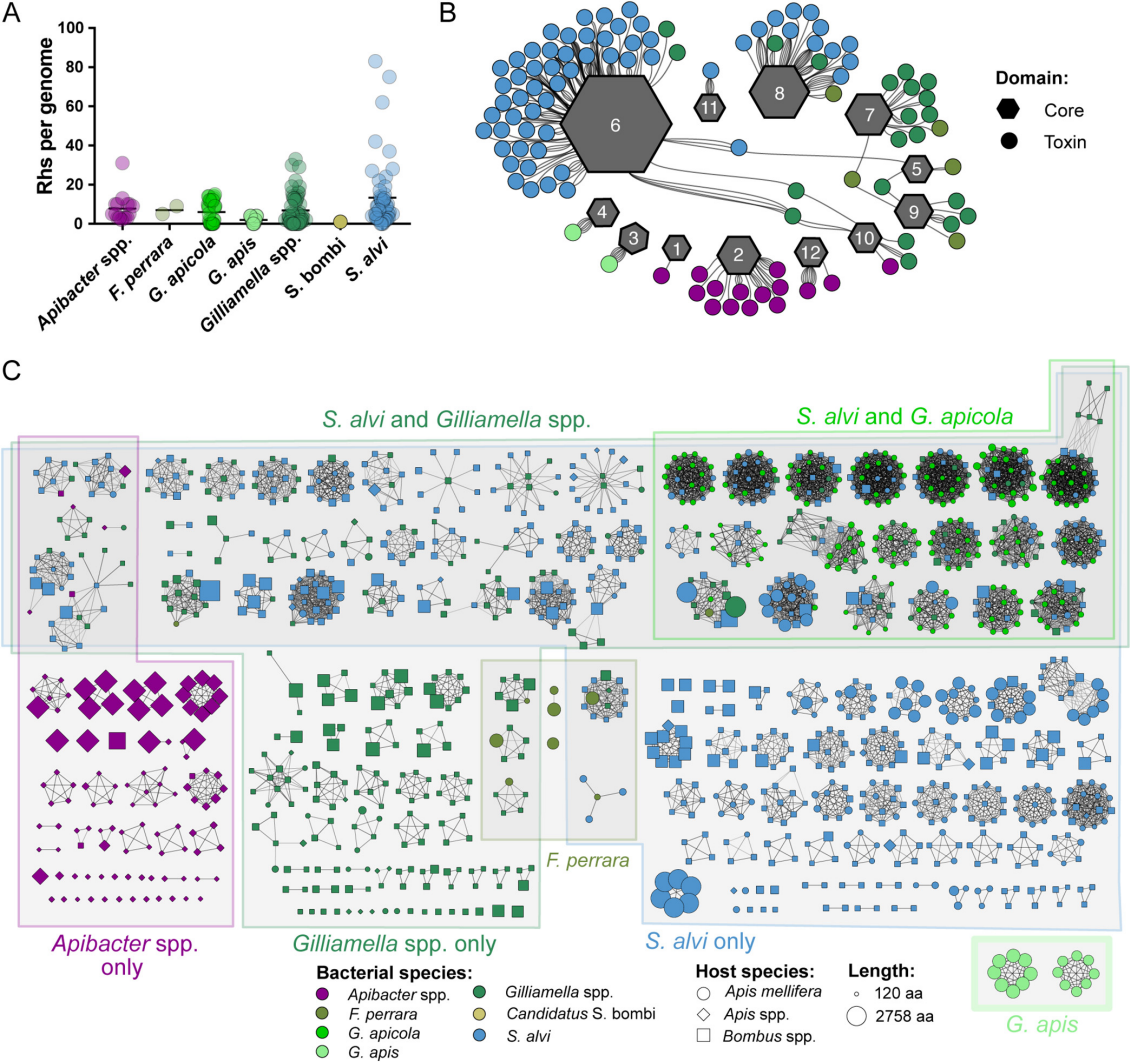
Rhs toxin diversity within bee gut symbionts. (A) Number of Rhs toxin domains per genome for each species cluster. C-terminal toxin domains were extracted from proteins containing Rhs repeat motifs and clustered. Final counts represent the number of proteins with ≥40% homology to representative toxin domains. (B) Network diagram showing relationships between Rhs toxin domains and core domains. Gray hexagons represent core domain clusters, with the size proportional to the number of proteins within the cluster. Circles represent toxin domain clusters, which are colored based on the bacterial species in which they are most often found. Edges connect core and toxin domains from the same protein. Not all toxin domains are shown, as many orphaned toxin domains do not include a large enough core domain to be assigned to a cluster. (C) Network diagram illustrating the composition of 208 toxin domain clusters. Each node represents a protein sequence from an isolate genome. Node color, shape, and size represent the bacterial species, the host from which it was isolated, and the protein length, respectively. Clusters are arranged based on whether they are found in multiple species clusters or restricted to one genus. Edges represent ≥40% amino acid identity over a minimum of 90 amino acids, as determined through all-versus-all protein BLAST.

Within the bee microbiota, all species that carry T6SSs also carry genes encoding Rhs toxins, though the number of toxin domains per genome varies (Fig. 3A). We found that each species utilizes only 1 to 4 distinct core domains, but a single core domain is often associated with many different toxin domains (Fig. 3B). Rhs proteins in *Gilliamella* spp. and F. perrara—both members of the family *Orbaceae*—contain similar core domains, which is consistent with the shared ancestry of these species. Full-length Rhs toxins with related core domains (Rhs core cluster 08) are present in *S. alvi*, *Gilliamella* spp., and *F. perrara* strains, suggesting that horizontal transfer of full-length toxin genes is possible, though transfer and retention of full-length genes appears to be rare. In a few cases, a single toxin domain was associated with multiple core domains, but only when the toxin was present in multiple species. Most toxins were found with only one core domain or were orphans that could not be assigned a core domain. Indeed, for 103 of the 208 toxin domain clusters identified, only orphaned toxin domains were present (Fig. 3C).

**FIG 4:**
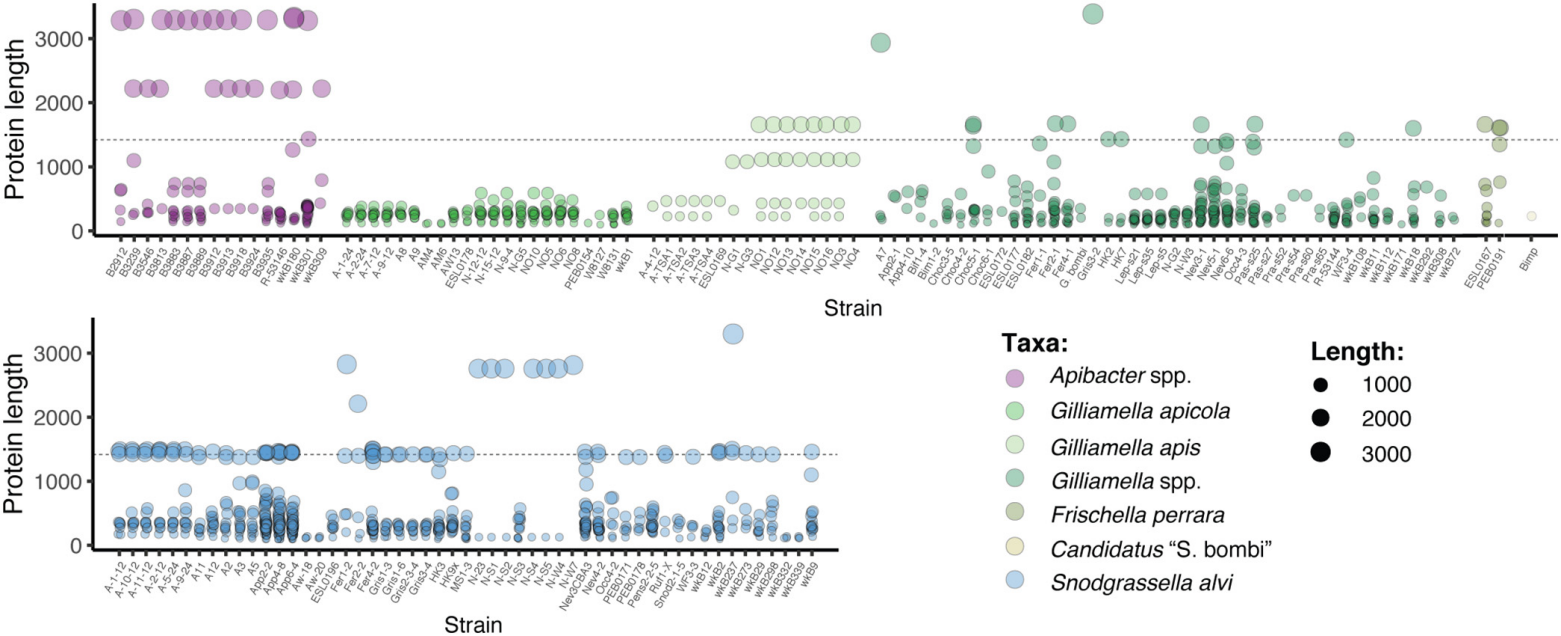
The genomes of bee gut microbes encode full-length Rhs toxins and orphaned C-terminal toxin domains. Each point represents a protein encoded by the genome of a bee gut isolate (*x* axis) containing a toxin domain homologous (≥40% identity over ≥90 amino acids) to a representative sequence from one of 208 toxin clusters. The size and color of each point represent the length of the protein and the species in which it is present. Full-length Rhs proteins are approximately 1,400 amino acids in length (indicated by the dashed line) and often include secretion domains. Smaller proteins are generally orphaned toxin domains. Proteins larger than 1,400 amino acids often contain Rhs repeat motifs similar to T6SS effectors but have N-terminal domains usually associated with other secretion systems, such as autotransporters and T9SSs.

Among bee gut isolates, full-length Rhs proteins are approximately 1,400 amino acids in length and consist of a conserved core domain, a C-terminal toxin domain, and a N-terminal secretion domain. In many bacteria, recombination between *rhs* genes with similar 5′ ends and different 3′ ends can result in displacement of the original 3′ end. This process, known as “C-terminal displacement,” sometimes forms long arrays of orphaned 3′ ends and immunity genes downstream of an intact gene (18, 19, 33) (see Fig. S5 in the supplemental material). Most bee gut taxa with *rhs* genes encode a mixture of full-length Rhs proteins and orphaned toxin domains (Fig. 4). On average, the genomes of *S. alvi* strains encode more full-length and orphaned Rhs toxins than other bee symbionts (Fig. 3A). The genomes of *S. alvi* isolates with the most orphaned toxin domains—particularly strains App2-2, App4-8, and App6-4—also encode more full-length Rhs proteins than other *S. alvi* strains (Fig. 4). The larger number of full-length genes in these isolates may boost the number of orphaned toxin domains by presenting more sites for recombination. Like *S. alvi*, *Gilliamella* spp. and *F. perrara* genomes encode both full-length toxins and orphaned toxin domains. However, unlike other *Gilliamella* spp., the genomes of *G. apicola* isolates encode only orphaned toxin domains, suggesting that Rhs toxins in *G. apicola* serve a purpose that does not require an N-terminal secretion domain to interact with the T6SS. Furthermore, most bee gut bacteria genomes encode a mixture of toxin domains found only within a single species cluster and toxin domains present in multiple genera. *G. apicola* is a notable exception, in that its genome encodes no Rhs toxins exclusive to *Gilliamella*. Instead, most of the Rhs toxins encoded by the genomes of *G. apicola* strains are orphaned toxin domains that are also found in *S. alvi* (Fig. 3C). In contrast, *Apibacter* spp. genomes encode few Rhs proteins that are also found in other genera. Surprisingly, many *G. apis* strains, which do not carry T6SS structural genes, carry genes encoding multiple Rhs toxins (Fig. 3A). Some of these toxin domains were identified in proteins with features that are not characteristic of T6SS effectors, discussed in more detail in the following section.

10.1128/mSystems.00063-21.5 FIG S5 Each *vgrG* gene in *S. alvi* wkB2 and *G. apicola* wkB7 is associated with a different set of accessory and effector genes. Each row depicts the organization of genes in one of the two VgrG loci in *S. alvi* wkB2 or nine VgrG loci in *G. apicola* wkB7. *vgrG* genes are labeled with the VgrG cluster to which they belong. Accessory and effector genes with significant homology to proteins in the NCBI Conserved Domains Database are colored and labeled with the conserved domain. Hypothetical genes with no conserved domain (CD) matches are shown in grey. Hypothetical genes with significant homology to one another are grey with colored outlines. Download FIG S5, TIF file, 1 MB. Copyright © 2021 Steele and Moran. 2021 Steele and Moran. https://creativecommons.org/licenses/by/4.0/ This content is distributed under the terms of the Creative Commons Attribution 4.0 International license.

**FIG 5:**
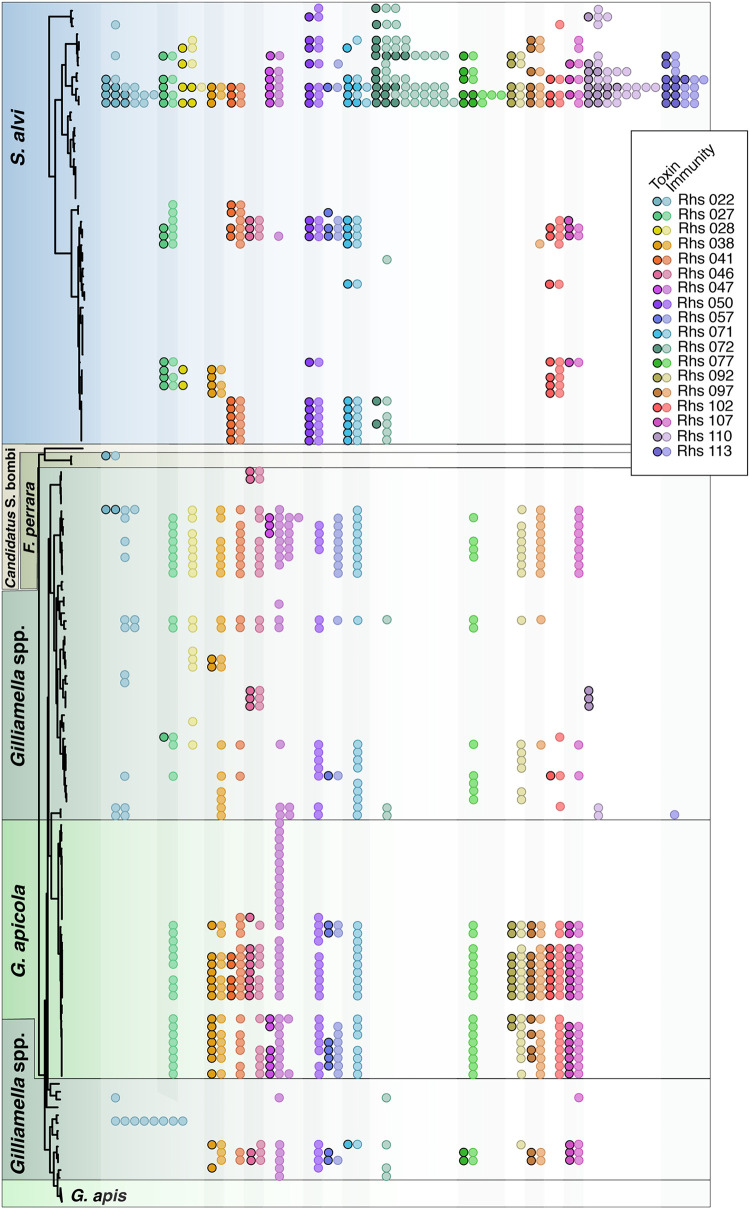
Immunity gene homologs are found with and without cognate Rhs toxins. Genomes of 198 bacterial isolates were searched for proteins homologous to 18 Rhs toxin domain and immunity protein pairs. Each row corresponds to a single genome in the *S. alvi* and *Gilliamella* spp. genome phylogenies on the left. The same phylogenies, with strain names, are shown in Fig. 2. Black outlines and shading delineate symbiont species. Each circle represents a toxin domain (black outline) or immunity protein (no outline) that shares ≥40% amino acid identity over ≥90% of its length with the reference sequence. Circle colors indicate toxin and immunity gene pairs. Some genomes contained multiple homologs, which are shown as multiple circles of the same color on a single row. In the rare instances in which a toxin was detected without its cognate immunity gene, either the immunity gene was not identified due to low homology to the reference sequence or the toxin was located at the end of a contig, which prevented annotation of the immunity gene.

As is the case for many T6SS effectors (4, 15, 34–37), immunity genes located near the 3′ end of each *rhs* gene encode proteins that protect bee symbionts from the effects of their own cognate toxins (28). Furthermore, many strains carry immunity gene homologs that may protect them from toxins produced by other members of the microbiota (Fig. 5). The genomes of *G. apicola* strains frequently encode toxin and immunity pairs homologous to those found in *S. alvi*, while other *Gilliamella* spp. tend to have many orphaned immunity genes without their cognate toxins.

### Similar toxin domains are associated with different types of secretion systems.

While Rhs toxins are common T6SS effectors, some proteins containing Rhs repeat motifs serve other functions. Many Rhs proteins encoded by *Apibacter* spp. genomes have N-terminal SpvB and TcdB domains instead of the PAAR domain characteristic of Rhs proteins secreted by T6SSs. Furthermore, the majority of *rhs* genes carried by *Apibacter* spp. are adjacent to genes encoding type A sorting-domain proteins of type IX secretion systems (T9SSs). Therefore, it seems probable that most, if not all, of the Rhs proteins in *Apibacter* spp. are associated with a T9SS and are not T6SS effectors. T9SSs allow gliding motility in Bacteroidetes spp. and are virulence factors for some pathogens (38). T9SSs may also secrete antibacterial polymorphic toxins (39), and it is possible that the Rhs proteins encoded by *Apibacter* spp. genomes play a role in interbacterial antagonism. However, this aspect of T9SS biology is not well understood and warrants further investigation.

In a few cases, we identified proteins containing filamentous hemagglutinin repeat domains and an extended signal peptide characteristic of type V secretion systems (T5SSs) that had C-terminal domains homologous to toxin domains of Rhs proteins. These proteins may be autotransporters used in contact-dependent growth inhibition. Toxin cluster 164 provides an example of this (see Fig. S6 in the supplemental material). Based on a maximum-likelihood phylogeny of these proteins, toxin domain 164 is associated with an autotransporter-like N-terminal domain in *S. alvi*, which has been horizontally transferred to *Gilliamella* spp. In other *Gilliamella* strains and in one *F. perrara* strain, this toxin domain is associated with an N-terminal domain containing Rhs repeats and is likely to have been horizontally transferred between *Gilliamella* and *F. perrara*. Toxin domain clusters 118 and 166 are also present as putative autotransporters in *S. alvi* genomes and as Rhs toxins in *Gilliamella* spp. As related toxin domains are found in bacteria not associated with bees, it is not clear whether this exchange of a toxin domain between an autotransporter and a Rhs family T6SS effector occurred within the bee gut microbiota. However, transfer of toxin domains between different polymorphic toxin systems illustrates one mechanism for acquiring novel effectors.

10.1128/mSystems.00063-21.6 FIG S6 Some toxin domains have been transferred between species and between secretion systems. A maximum-likelihood phylogeny was constructed for an alignment of proteins from toxin domain cluster 164, using RAxML with the WAG likelihood model of amino acid replacement and 1,000 bootstraps. Grey circles mark nodes with ≥70% bootstrap support. Strain names are colored by genus, triangle symbols along the right edge indicate different N-terminal domains, and white and grey squares mark strains from different host genera. Rhs core 07 and 09 domains were assigned based on homology to representative sequences of core domain clusters. Putative autotransporter domains were identified using the NCBI Conserved Domain Database. Download FIG S6, TIF file, 2.9 MB. Copyright © 2021 Steele and Moran. 2021 Steele and Moran. https://creativecommons.org/licenses/by/4.0/ This content is distributed under the terms of the Creative Commons Attribution 4.0 International license.

### Patterns of VgrG diversification are lineage specific.

While Rhs proteins are common T6SS toxins, many other effectors are secreted by T6SSs. VgrG is a structural component of T6SSs and provides a loading site for many T6SS effectors (40–42). The VgrG C terminus is required for effector transport and is typically associated with a specific effector and its cognate immunity gene (19, 40–42). Like Rhs toxins, *vgrG* genes can diversify through C-terminal displacement. Thus, we used VgrG diversity as a proxy for the diversity of effectors that bind to VgrG. We identified 977 proteins with VgrG motifs in our set of bee gut isolate genomes, which clustered into 13 groups. 663 proteins were greater than 500 amino acids in length and shared at least 40% amino acid identity with a representative sequence from one of the 13 clusters.

**FIG 6:**
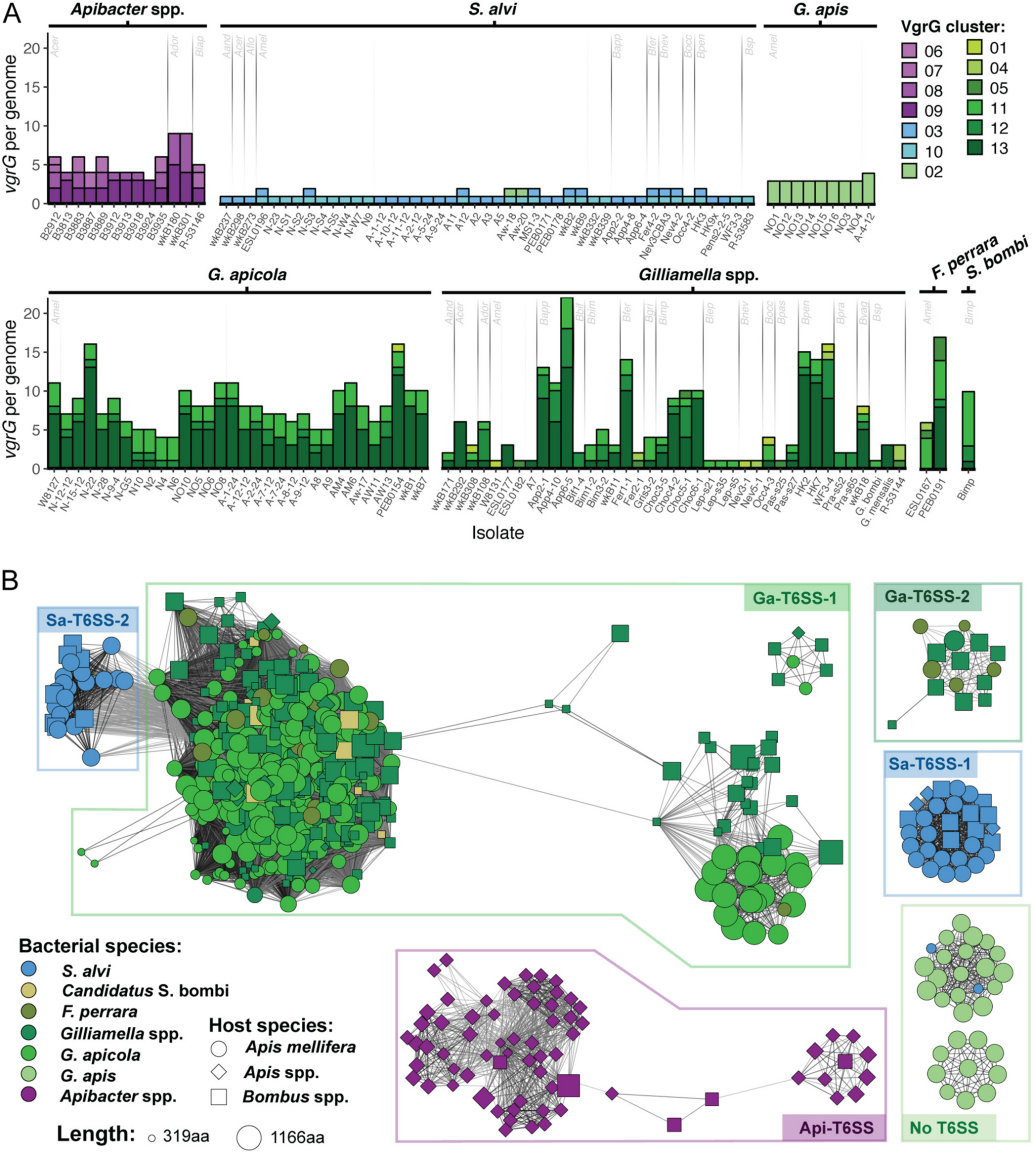
VgrG diversity within bee gut symbionts. (A) Total number of *vgrG* genes per isolate (*x* axis). Colors of bars represent VgrG clusters sharing ≥40% amino acid identity. Isolates are grouped by bacterial taxa. For taxa present in multiple hosts, gray lines separate isolates from different host species, with the host species indicated as follows: *Aand*, Apis andreniformis; *Acer*, Apis cerana; *Ador*, Apis dorsata; *Amel*, Apis mellifera; *Bapp*, Bombus appositus; *Bbif*, Bombus bifarius; *Bbim*, Bombus bimaculatus; *Bfer*, Bombus fervidus; *Bgri*, Bombus griseocollis; *Bimp*, Bombus impatiens; *Blep*, Bombus lepidus; *Bnev*, Bombus nevadensis; *Bocc*, Bombus occidentalis; *Bpas*, Bombus pascuorum; *Bpen*, Bombus pensylvanicus; *Bpra*, Bombus pratorum; *Bvag*, Bombus vagans; *Bsp*, unspecified *Bombus* sp. Strains with no *vgrG* genes are not shown. (B) Homology network for 663 VgrG proteins encoded by bee gut bacteria genomes. An all-versus-all BLAST search was used to identify homologous proteins. Each node represents a single protein sequence from a bee gut isolate, with color, shape, and size of the node indicating the isolate’s species, the host from which it was isolated, and the protein length, respectively. Edges represent ≥40% amino acid identity over ≥90% of the protein length. Boxes around clusters group proteins associated with particular T6SS loci.

The average number of VgrG proteins differs among species. *S. alvi* strain genomes encode only one VgrG protein per T6SS, whereas *Apibacter* and *Gilliamella* spp. genomes frequently encode multiple VgrG proteins (Fig. 6A and Fig. S5). Furthermore, the number of *vgrG* genes per *Gilliamella* strain differs among isolates from different host species, suggesting that host factors that allow loss of T6SSs in bumble bee—but not honey bee—microbiotas may also allow reductions in effector diversity.

VgrG proteins are a structural component of the T6SS and are therefore likely to share an evolutionary history with other structural genes. *S. alvi* VgrG proteins form two clusters, which are associated with Sa-T6SS-1 and Sa-T6SS-2, respectively (Fig. 6B). The genes that encode these proteins are associated with the T6SS loci. VgrG proteins found in *Gilliamella* spp., *F. perrara*, and “*Candidatus* Schmidhempelia bombi” form four clusters that are likely derived from a common ancestor associated with Ga-T6SS-1. Most *vgrG* genes in members of the family *Orbaceae* occur in satellite loci, far from the genes encoding the structural components of the T6SS. In some strains, one copy of *vgrG* is located within the Ga-T6SS-1 locus (Fig. S3). While *F. perrara* and “*Candidatus* Schmidhempelia bombi” resemble *Gilliamella* spp. in VgrG diversity, they differ in which VgrG cluster is most abundant, suggesting expansion of different VgrG families in different lineages (Fig. 6A and C). Two VgrG clusters are associated with Ga-T6SS-2; one is found only in *Gilliamella* spp., and one, like Ga-T6SS-2, shows evidence of horizontal transfer between *Gilliamella* spp. and *F. perrara* (Fig. 6B). Four VgrG clusters are associated with a T6SS in *Apibacter* spp. and also appear to share an ancestor. The *Apibacter* spp. *vgrG* genes are found in satellite loci. Surprisingly, there is a conserved VgrG-like protein in some *G. apis* strains, whose genomes encode no other T6SS structural components. These *G. apis* strains, including isolates from two different host populations, carry multiple copies of *vgrG*, which are located in clusters of genes similar to the auxiliary VgrG clusters found in other Gammaproteobacteria. Fragments of this *vgrG*-like gene are also present in the genomes of two *S. alvi* isolates (Fig. 6B).

**FIG 7:**
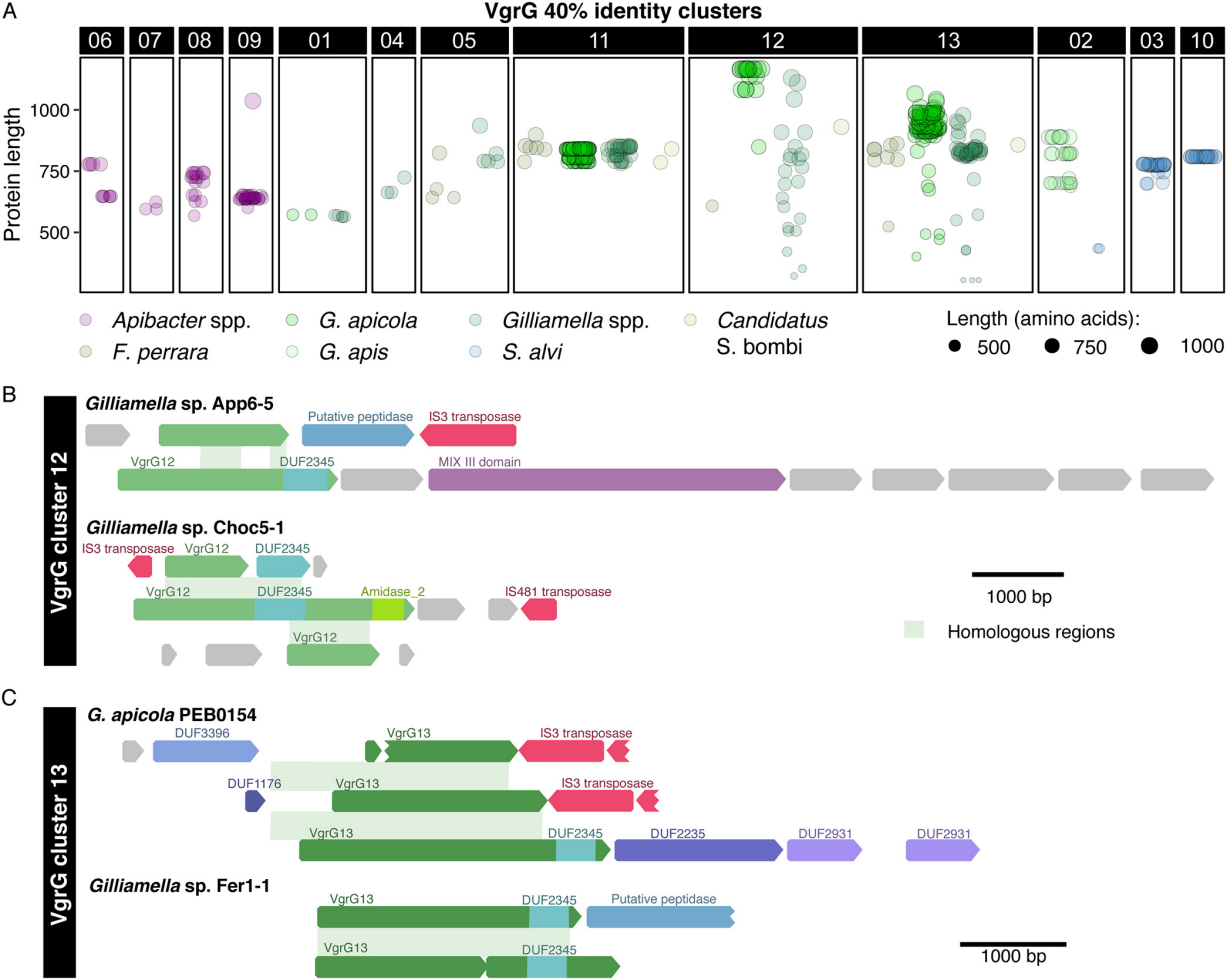
Pseudogenization of *vgrG* in clusters 12 and 13. (A) Variation in the lengths of individual VgrG proteins provides insight into the process of diversification. VgrG proteins are split by cluster (upper *x* axis) and across bacterial species clusters for clusters found in multiple species (lower *x* axis). Colors represent bacterial species, while the size of each point indicates protein length. (B) Multiple *vgrG* genes (green) homologous to the VgrG 12 cluster show signs of disruption by mobile genetic elements (red) or nonsense mutations (indicated by truncation of the gene relative to other genes in the cluster). Each row depicts a single VgrG locus. Accessory or effector genes with identifiable conserved domains are indicated. Hypothetical genes with no known domains are shown in gray. Green shading connects homologous regions of different VgrG loci from the same genome. (C) As in VgrG 12, multiple genes within the VgrG 13 cluster have been disrupted by nonsense mutations and mobile genetic elements.

Homologous VgrG proteins show considerable variation in length, which could reflect degradation of unneeded copies after gene duplication or formation of displaced fragments due to illegitimate recombination. VgrG fragments were far more common in the five bee gut-associated *Orbaceae* (*Gammaproteobacteria*) spp. than in *Apibacter* spp. (*Bacteroidetes*) or *S. alvi* (Betaproteobacteria) (Fig. 7A). Most of the VgrG proteins in the *Orbaceae* taxa belong to one of three 40% identity clusters. The truncated proteins in clusters 12 and 13 appear to be the result of pseudogenization through a combination of nonsense mutations and insertion of mobile genetic elements into *vgrG* genes (Fig. 7B and C). In contrast, cluster 11, which is found in the same bacterial genera and overlaps with cluster 13 in the homology network, is far less variable.

VgrG proteins in *S. alvi* are highly conserved and show little divergence, duplication, or fragmentation (Fig. 7A), despite cluster 10 being related to the highly diversified VgrG found in *Gilliamella* spp. (Fig. 6B). As in *S. alvi*, VgrG proteins in *G. apis* vary little in length and sequence (Fig. 7A), but this could reflect high relatedness of *G. apis* isolates carrying *vgrG* genes. VgrG proteins in *Apibacter* spp. differ more than those in *S. alvi*, but differ far less in length than do *Gilliamella* spp. proteins, suggesting that *vgrG* genes in *S. alvi* and *Apibacter* spp. are less likely to undergo paired homologous and illegitimate recombination events that result in orphaned domains. If so, the frequency of C-terminal displacement is not consistent across different effectors, as *S. alvi*, the species with the highest rate of Rhs C-terminal displacement, also has the lowest rate of VgrG diversification (see Fig. S7 in the supplemental material).

10.1128/mSystems.00063-21.7 FIG S7 Bacterial lineages specialize in different mechanisms of toxin diversification. The number of Rhs proteins per genome is plotted against the number of VgrG proteins per genome. Colors indicate different bacterial species clusters; shapes indicate different host species. Download FIG S7, TIF file, 0.2 MB. Copyright © 2021 Steele and Moran. 2021 Steele and Moran. https://creativecommons.org/licenses/by/4.0/ This content is distributed under the terms of the Creative Commons Attribution 4.0 International license.

## DISCUSSION

The bee gut microbiota provides interesting parallels with other host-associated microbial communities. For example, half of the *Bacteroidales* strains isolated from the human gut—about one fourth of the bacteria in the intestine—carry genes encoding T6SSs (3). In honey bees (A. mellifera), *S. alvi* and *G. apicola* typically comprise more than half of the bacteria in the ileum (equivalent to the small intestine) (43), and almost all strains carry genes encoding at least one T6SS. Another system with similarities to the bee gut microbiota is the symbiotic relationship between Aliivibrio fischeri and bobtail squid. The diversity of A. fischeri is high in seawater, as diversity of *G. apicola* and *S. alvi* is high in honey bee hives overall (29), but light organ crypts within individual squid (44) and the guts of individual bees (21, 30) are dominated by single strains or clusters of closely related strains. T6SSs help to exclude incompatible V. fischeri strains from crypt spaces in bobtail squid (6, 45). Similarly, Bacteroides fragilis uses a T6SS to displace competitors in the mouse gut (2, 4, 5, 34). T6SSs likely allow bacteria to dominate niches in the bee gut as well.

T6SSs may have contributed to the establishment of features seen in the modern bee gut microbiota. For example, bee gut bacteria participate in cooperative metabolic interactions (26, 46, 47), which may be facilitated by T6SS-mediated antagonism that excludes cheaters (48). Additionally, *S. alvi* and *G. apicola* cocolonize the bee ileum, where they form organized layers (43). Spatial separation is likely enforced in part by T6SSs, although environmental gradients within the gut, including concentrations of oxygen (49) and host-produced antimicrobial peptides (50), may also contribute.

We observed similar host-specific trends in T6SS retention and toxin diversification for both *S. alvi* and *Gilliamella* spp. Lower bacterial diversity in bumble bee hosts relative to that in honey bee hosts (21, 27) may help to explain why bumble bee isolates sometimes lose their T6SSs and can differ drastically in the number of T6SS effectors encoded in their genomes. A smaller population size also increases the effect of genetic drift, allowing genotypes with potentially deleterious traits (e.g., the loss of T6SS genes) to dominate. Pesticides and antibiotics reduce the diversity of honey bee gut symbionts and increase vulnerability to bacterial pathogens (51, 52), and these short-term disruptions could affect T6SS-mediated antagonism within the microbiota. Social and behavioral factors that alter microbiota diversity within human populations (e.g., antibiotic use and diet) might similarly affect the evolution of interbacterial antagonism among human gut bacteria.

Within the bee microbiota, T6SS loss and retention is connected to symbiont speciation. This is most apparent in Gilliamella, which has split into multiple species in honey bees, most notably *G. apicola* and *G. apis*. These species have very similar 16S rRNA gene sequences but differ in their metabolic capabilities (29, 53). Here, we show that *G. apicola* isolates almost always carry genes encoding a single, highly conserved T6SS, whereas all *G. apis* isolates lack T6SS genes. *G. apicola* and *G. apis* cooccur within the guts of individual bees (29) despite their apparent differences in capacity for interstrain antagonism, suggesting that these closely related species occupy different niches within the gut.

While host-specific selective pressures may affect the retention of T6SS structural genes, effector diversity seems to be driven by factors specific to bacterial lineages. T6SS genes are not transferred between cooccurring members of different phyla, but Rhs toxin domains—and presumably their cognate immunity genes—are exchanged among *S. alvi* (*Betaproteobacteria*) and *Gilliamella* (*Gammaproteobacteria*) isolates. As we reported previously (28), *S. alvi* strains carrying genes encoding Sa-T6SS-1—with or without Sa-T6SS-2—tend to have many Rhs toxins, while strains lacking Sa-T6SS-1 have few or none. In contrast, no strong relationship is apparent between T6SS loci and Rhs diversity in *G. apicola*. Furthermore, the genomes of *G. apicola* strains encode only orphan toxin domains that are homologous to proteins found in multiple species clusters. As these proteins lack secretion domains necessary to associate with the T6SS, acquisition of orphaned toxin domains along with linked immunity genes may be a defensive strategy in *G. apicola*, similar to that used by *Bacteroidales* species in the human gut (34). The genomes of many *S. alvi*, *Gilliamella* spp., and *F. perrara* isolates encode a mixture of full-length Rhs proteins and orphaned C-terminal domains, indicating that Rhs diversification through C-terminal displacement occurs in multiple bacterial lineages. In species with full-length Rhs proteins, orphaned toxin domains may serve as a reservoir of toxin diversity, potentially becoming reattached to a secretion domain through homologous recombination in the future (20).

Diverse sets of effector proteins provide multiple advantages. In Pseudomonas aeruginosa, T6SS effectors work synergistically to kill target cells, but also have different optimal environmental conditions, allowing cells to remain competitive in a range of environments (54). For microbes in more constant (e.g., host) environments, acquisition of novel toxins provides a way to overcome the immunity of sister cells or community members that have acquired immunity genes. Although *S. alvi*, *Gilliamella* spp., *F. perrara*, and “*Candidatus* Schmidhempelia bombi” have T6SSs belonging to the same subtype (T6SS^i2^) (31), they have different modes of toxin diversification. Diversification of VgrG and Rhs proteins through C-terminal displacement in *Gilliamella* spp., *F. perrara*, and “*Candidatus* Schmidhempelia bombi” resembles that described previously for other *Gammaproteobacteria* (19). Furthermore, these species carry genes encoding related VgrG proteins, but different VgrG clusters have diversified in each genus. The extensive diversification of Rhs proteins in *S. alvi* suggests selective pressure to acquire and deploy novel toxins (28), but *S. alvi vgrG* genes do not undergo C-terminal displacement. Further comparison of T6SS effector diversification across species may provide insight into the genetic factors that promote or constrain the expansion of particular effector families, which could have significant ramifications for how T6SSs participate in the evolution of bacterial communities.

### Conclusion.

A small number of bacterial species are present within the guts of honey bees and related social bee species and have codiversified with their hosts. In this study, we used comparative genomics to examine how community-wide evolutionary pressures affect the diversification of T6SSs and T6SS effectors within multiple members of the bee gut microbiota. Many bee gut bacteria carry genes encoding highly conserved T6SSs, present at the onset of their association with bee hosts and retained by most, but not all, strains. T6SS loss is connected to both host and bacterial speciation. Additionally, gut bacteria carry genes encoding numerous effector proteins and demonstrate lineage-specific mechanisms for effector diversification. Future work is likely to identify many ways in which T6SS-mediated antagonism has influenced the bee microbiota, including effects on strain diversity, speciation, spatial organization, and cooperation.

## MATERIALS AND METHODS

### TssB, TssC, and TssH phylogenies.

Phylogenies of TssB, TssC, and TssH proteins, which have previously been used for inferring phylogenetic relationships of distantly related T6SSs (31), were used to assign T6SSs from bee gut isolates to previously described T6SS subfamilies (T6SS^i1^, T6SS^i2^, T6SS^i3^, T6SS^i4a^, T6SS^i4b^, T6SS^i5^, and T6SS^iii^), excluding T6SS^ii^, which is found only on the Francisella pathogenicity island (1, 55). The SecReT6 database (56) was used to identify representative T6SSs from each subfamily, and the corresponding protein sequences were downloaded from the NCBI RefSeq database (see Table S2 in the supplemental material). Proteins were aligned using the MUSCLE extension in Geneious v10.1.3 (57), and protein phylogenies were generated using RAxML v8.2.12 (58) with the LG likelihood model of amino acid substitution and 1,000 bootstraps. The phylogenies were visualized using the Interactive Tree of Life (iTOL) viewer (59).

### Genome phylogenies.

Genomes analyzed in this study are listed in Table S3 in the supplemental material. Snodgrassella alvi, *Gilliamella* spp., Bartonella apis, Parasaccharibacter apium, and *Apibacter* spp. protein and genomic nucleotide sequences were downloaded from the NCBI RefSeq database. OrthoFinder v2.3.7 (60, 61) was used to identify orthogroups for *S. alvi*, *Gilliamella* spp., and *Apibacter* spp. isolate genomes and to build species phylogenies for each from single-copy gene trees, which were visualized using the iTOL viewer (59).

10.1128/mSystems.00063-21.10 TABLE S3 Bee gut isolate genomes analyzed in this study. Download Table S3, PDF file, 0.2 MB. Copyright © 2021 Steele and Moran. 2021 Steele and Moran. https://creativecommons.org/licenses/by/4.0/ This content is distributed under the terms of the Creative Commons Attribution 4.0 International license.

### T6SS phylogenies.

Nucleotide sequences for representative T6SS loci were extracted from genomic sequences of *S. alvi* wkB2, Gilliamella apicola wkB1, *Gilliamella* sp. Choc5-1, Apibacter adventoris wkB180, Frischella perrara PEB0191, and “*Candidatus* Schmidhempelia bombi” Bimp. Proteins encoded by genes within each locus were identified by using NCBI BLAST+ v2.6.0 tblastx to search protein sequences for each genome assembly using the nucleotide sequence as a query, and then NCBI BLAST+ v2.6.0 blastdbcmd was used to extract the corresponding protein sequences (62).

A list of taxonomically diverse genomes encoding potential homologs to T6SS proteins from bee gut isolates was obtained by searching for homologous nucleotide sequences within the NCBI Representative Prokaryote Representative Genomes Database (283,128 sequences, last updated 28 October 2019). Genomes were included if they had matches (minimum 70% coverage and 40% identity) to at least three proteins from a representative T6SS locus. NCBI Batch ENTREZ was used to locate RefSeq protein files for each genome assembly. Bee gut isolate and outgroup proteins were converted into two BLAST databases. NCBI BLAST+ v2.6.0 blastp (62) was used to identify possible homologs to the representative bee gut T6SS proteins with a minimum 20% coverage and 20% amino acid identity. MUSCLE v3.8.31 (63) was used to make alignments for each set of homologs. Alignments were concatenated and manually curated in Geneious v10.1.3 (57). RAxML v8.2.12 was used to identify the optimal amino acid replacement model for each alignment and to generate maximum-likelihood phylogenies with 1,000 bootstraps. Phylogenies were visualized using the iTOL viewer (59).

In our analysis, bacterial genomes were considered to encode a T6SS if genes encoding all 13 of the T6SS structural proteins were present, with the following exceptions. T6SS loci with one missing gene were considered complete if the missing gene was located at the end of a contig. The *Apibacter* T6SS, which belongs to the T6SS^iii^ clade and is not as well characterized as the T6SSs found in Proteobacteria, was considered complete despite the absence of homologs to *tssQ* and *tssR*. From our observations, T6SS loss on the evolutionary timescale relevant to this study typically involves deletion of the entire T6SS locus or loss of part of the locus followed by pseudogenization of the remaining genes (28).

### Effector clustering.

A database of protein sequences was prepared that included all of the annotated proteins from each bee gut isolate genome. HMMER v3.1 (64) was used to identify proteins that matched either the TIGRFAM hidden Markov model (HMM) profile TIGR03696 or an HMM profile prepared from an alignment of previously identified Rhs core domains from *S. alvi* proteins. The TIGR01646 HMM profile was used to identify VgrG proteins. Protein sequences were extracted using blastdbdmd and aligned using MUSCLE v3.8.31. Rhs alignments were visually inspected in Geneious v10.1.3, and the conserved DPxG motif, which is present at the end of the conserved core region of Rhs proteins (15, 18), was used to identify C-terminal toxin domains. Protein sequences lacking this conserved region were removed from the alignment. C-terminal toxin domains were extracted from the alignment and exported for clustering. This was preferable to using the whole Rhs protein in analyses, as *rhs* genes are known to undergo recombination (18).

VgrG proteins and Rhs C-terminal domains were clustered into protein families using CD-HIT v4.6.8 (65) with a 40% identity cutoff. Representative sequences were selected for each cluster, and blastp was used to search for homologous proteins with ≥40% amino acid identity and an amino acid alignment length of ≥90 for Rhs toxin domains and ≥300 for VgrG proteins in bee gut isolate genomes. To avoid inclusion of partial sequences, 179 VgrG proteins located at the ends of contigs were excluded from analysis of VgrG length. A custom bash script was used to evaluate the abundance of each toxin domain and to join these data to isolate metadata. All-versus-all blastp searches were performed to generate homology networks, which were visualized in Cytoscape v3.3.0 (66). RAxML was used to generate maximum-likelihood phylogenies for individual effector clusters, which were visualized using the iTOL viewer. VgrG loci were compared using Mauve v2.3.1 (67, 68). Diagrams of VgrG loci in *S. alvi* wkB2 and *G. apicola* wkB7 were generated using Geneious. Conserved domains were identified using the NCBI Conserved Domains Database search tool (69).

To examine the cooccurrence of Rhs toxin and immunity genes in the bee microbiota, representative proteins were chosen for 18 of the 208 toxin domain clusters identified in this study. Putative immunity genes were identified based on proximity to the 3′ end of the toxin gene. For each toxin and immunity protein, protein BLAST was used to identify homologs with ≥40% amino acid identity and ≥90% coverage within a database comprised of all protein sequences from the 198 isolate genomes included in this study.

Scripts created to assist in these analyses are available at https://github.com/misteele/bee-gut-T6SS-scripts.
