# Supplementary figures and images for: Evolution of Interbacterial Antagonism in Bee Gut Microbiota Reflects Host and Symbiont Diversification
Source: mSystems. 2021 May 11;6(3):e00063-21. doi: 10.1128/mSystems.00063-21 (PMC8125069; doi:10.1128/mSystems.00063-21)

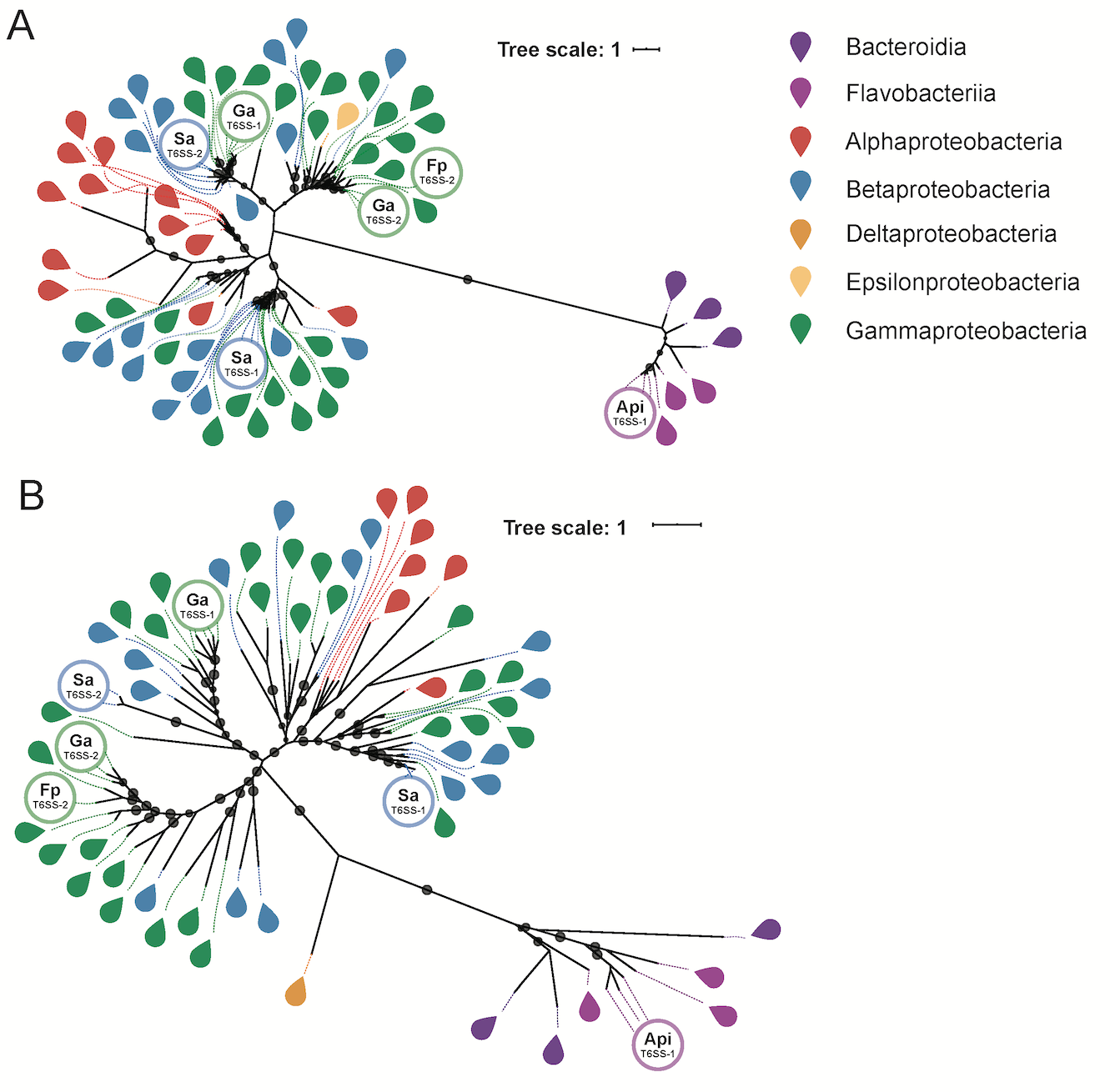

Supplement: FIG S1 [file mSystems.00063-21-sf001.tif]

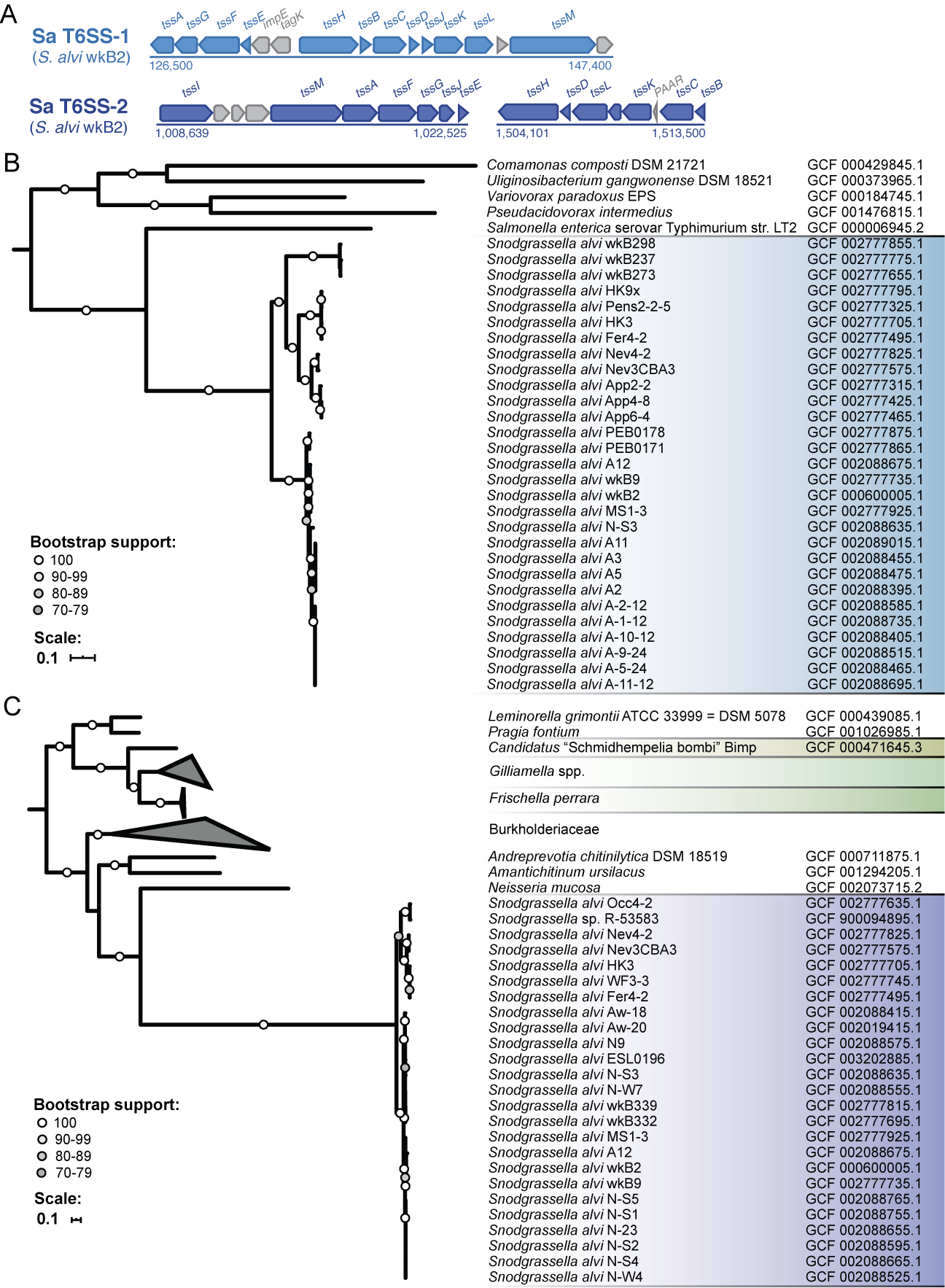

Supplement: FIG S2 [file mSystems.00063-21-sf002.tif]

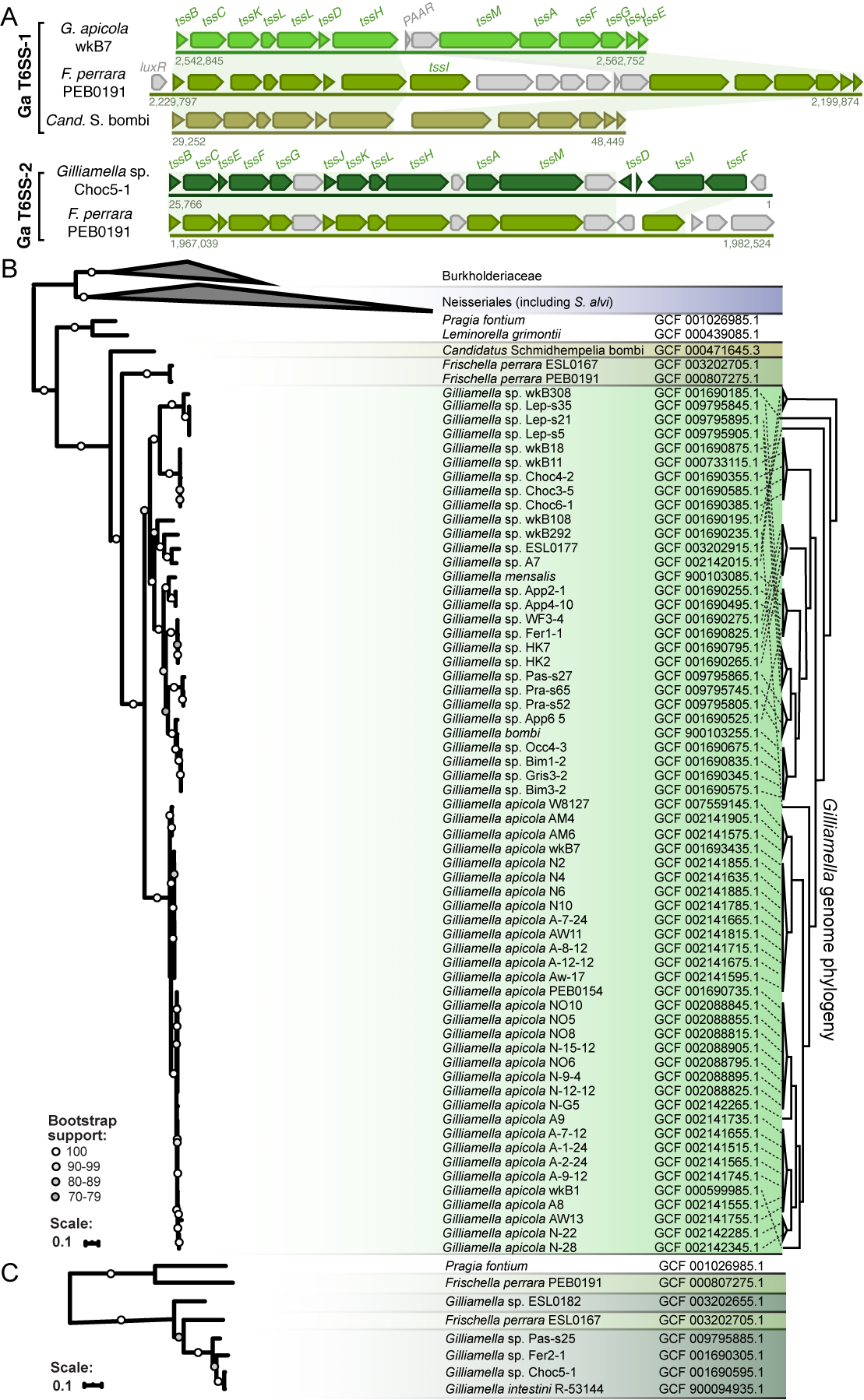

Supplement: FIG S3 [file mSystems.00063-21-sf003.tif]

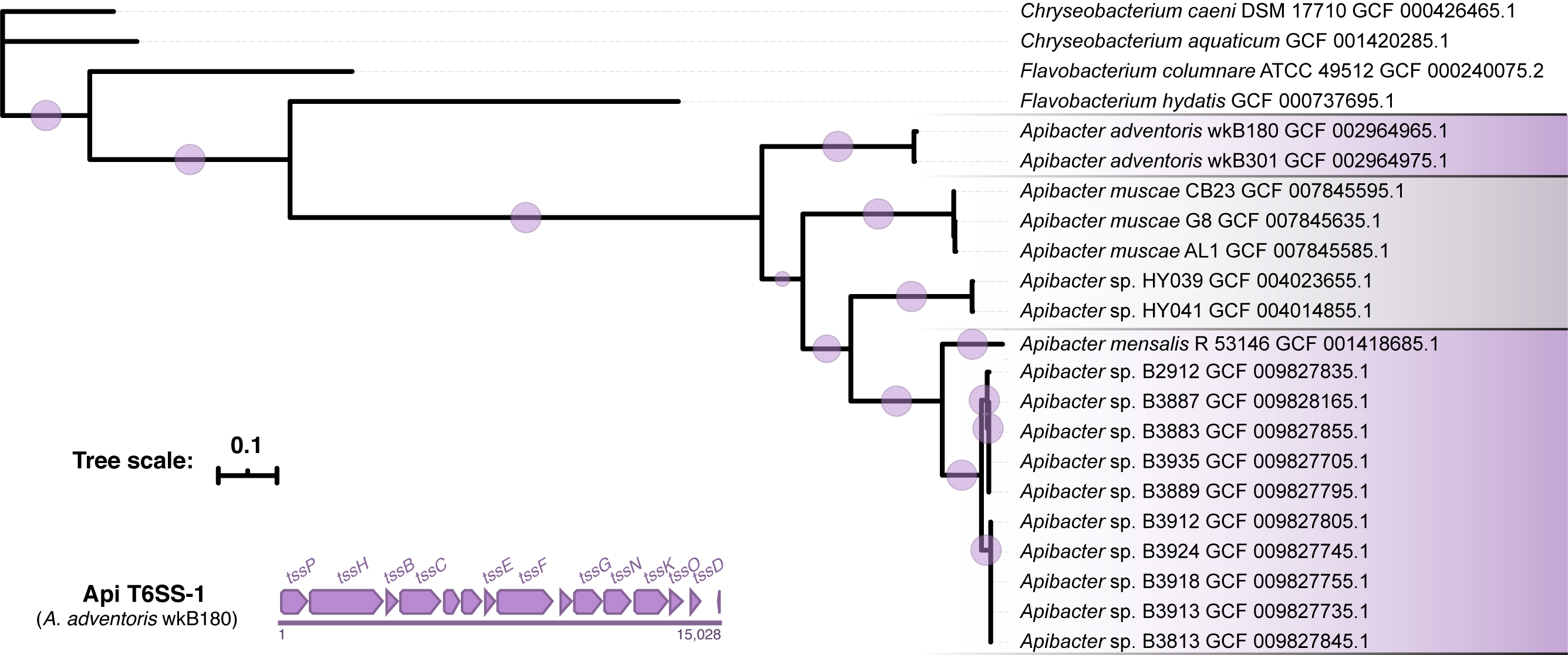

Supplement: FIG S4 [file mSystems.00063-21-sf004.tif]

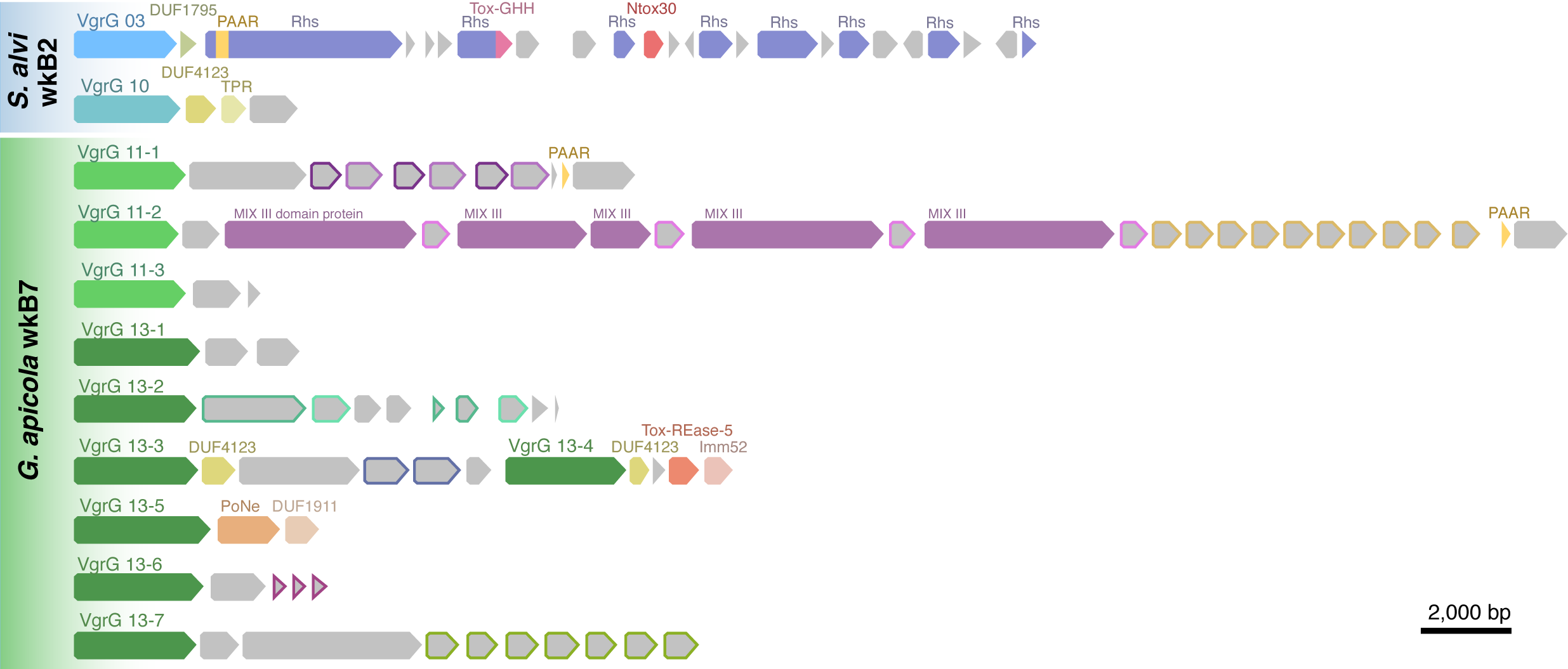

Supplement: FIG S5 [file mSystems.00063-21-sf005.tif]

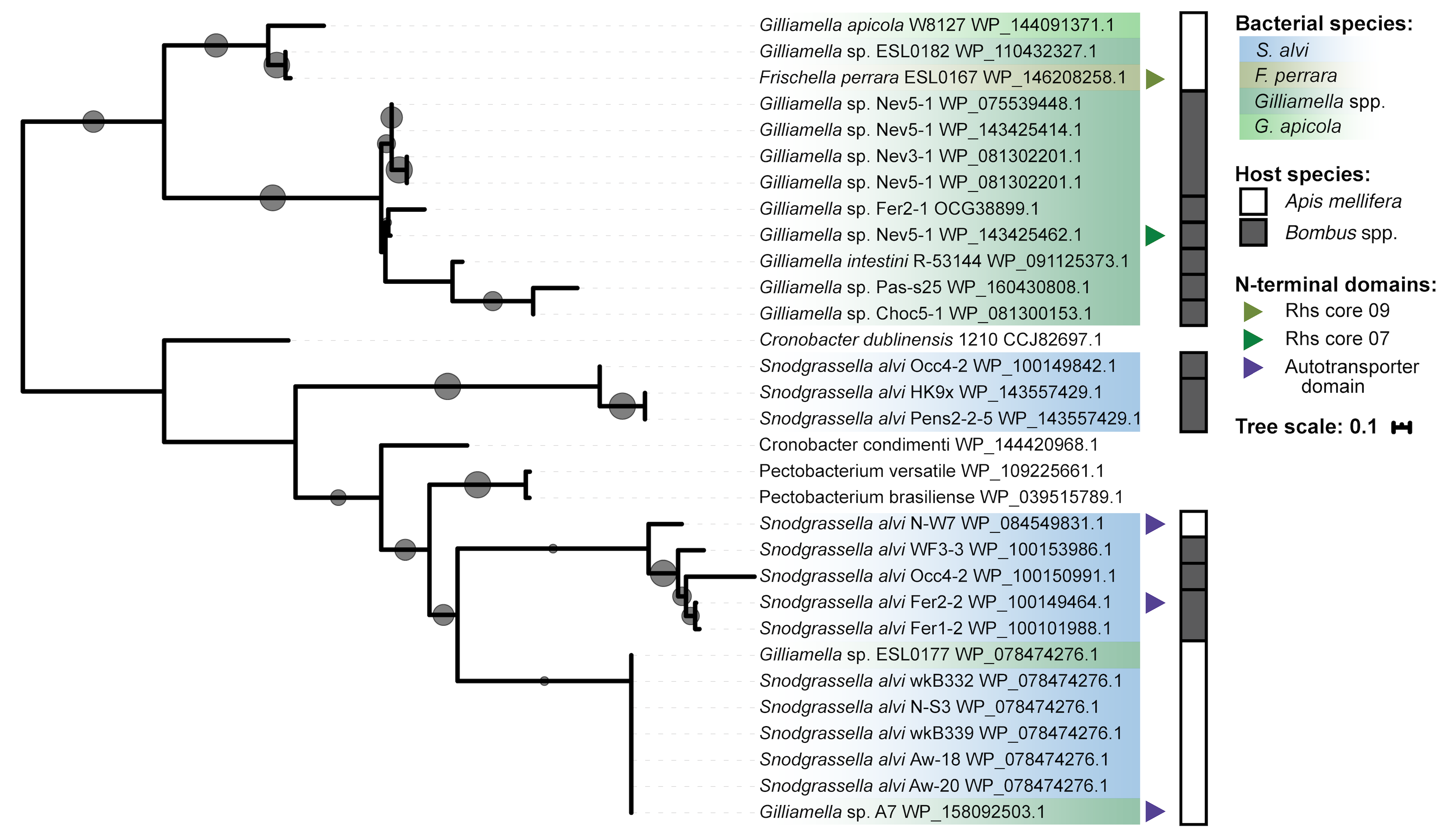

Supplement: FIG S6 [file mSystems.00063-21-sf006.tif]

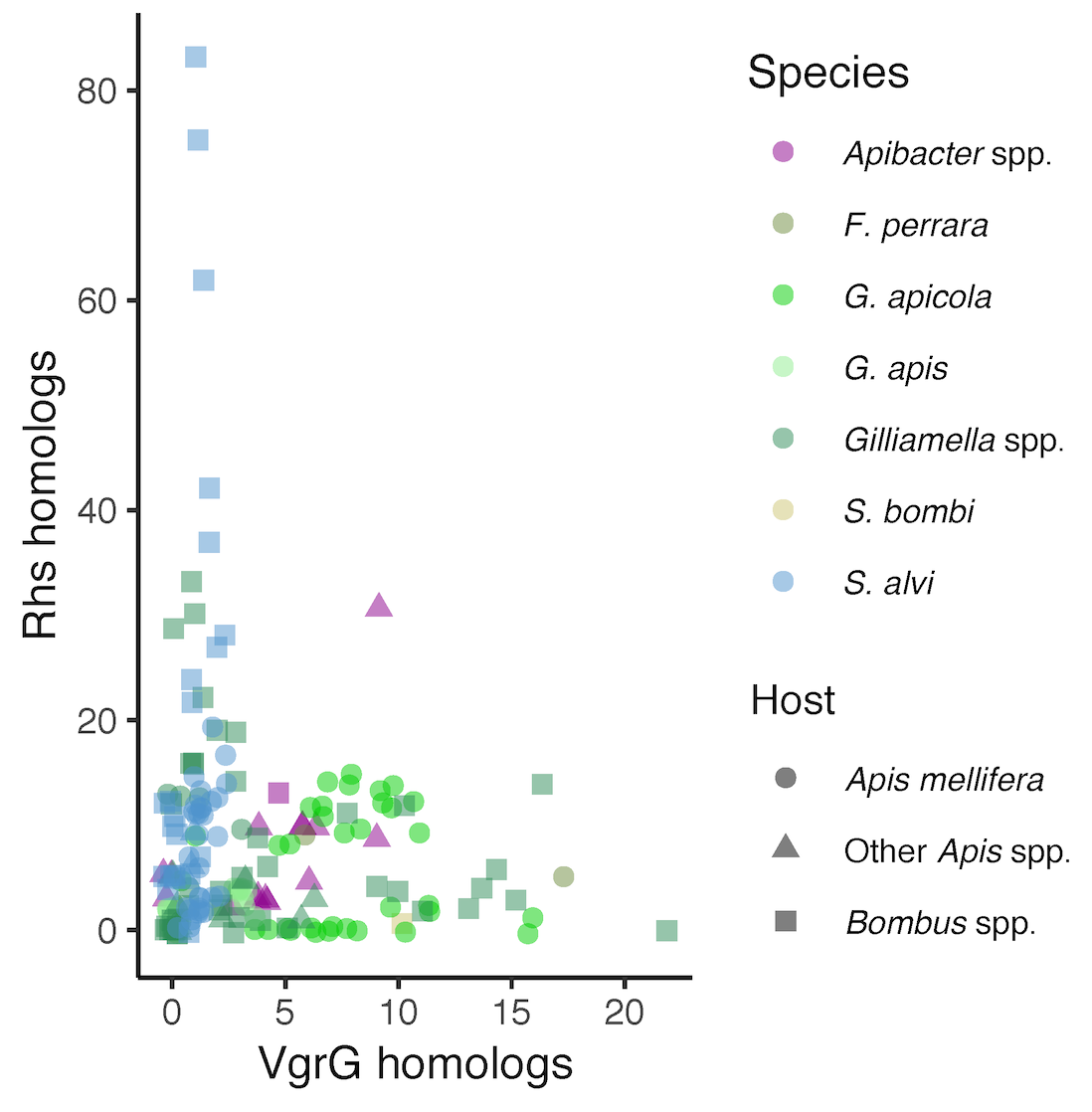

Supplement: FIG S7 [file mSystems.00063-21-sf007.tif]
