# Supplementary material for: Evolution of Interbacterial Antagonism in Bee Gut Microbiota Reflects Host and Symbiont Diversification
Source: mSystems. 2021 May 11;6(3):e00063-21. doi: 10.1128/mSystems.00063-21 (PMC8125069; doi:10.1128/mSystems.00063-21)
Supplement: TABLE S1 [file mSystems.00063-21-st001.pdf]

Table S1. Conserved domains found in Rhs toxins from genomes of bee gut symbionts.

Toxin domain	CDD top hit	Accession	E-value	Copies	Genus (full length)	Genus (fragment)	Rhs core domains
Rhs_tox_001				3	*Apibacter*	*Apibacter*	Rhs_core _12
Rhs_tox_002				1		*Snodgrassella*	
Rhs_tox_003				1		*Apibacter*	
Rhs_tox_004	DUF2778	pfam10908	2.91E-09	1		*Apibacter*	
Rhs_tox_005				1		*Apibacter*	Rhs_core _2
Rhs_tox_006				1		*Apibacter*	
Rhs_tox_007				1		*Apibacter*	
Rhs_tox_008				6		Multiple	
Rhs_tox_009	DUF4258	pfam14076	0.00014	2		*Apibacter*	
Rhs_tox_010				1		*Apibacter*	
Rhs_tox_011				1		*Apibacter*	
Rhs_tox_012				5	*Apibacter*	*Apibacter*	Rhs_core _1
Rhs_tox_013				1		*Apibacter*	
Rhs_tox_014				1		*Apibacter*	
Rhs_tox_015	ZnMc_serralysin_like	cd04277	8.13E-06	3		*Apibacter*	
Rhs_tox_016				1		*Apibacter*	
Rhs_tox_017				2		*Apibacter*	
Rhs_tox_018				1		*Apibacter*	
Rhs_tox_019				2		*Apibacter*	
Rhs_tox_020				8	*G. apis*		Rhs_core _3
Rhs_tox_021	Ntox47	pfam15540	9.67E-19	2		*Apibacter*	
Rhs_tox_022	Endonuclea_NS_2	pfam13930	8.41E-07	14		*Snodgrassella*<br>*Gilliamella*<br>*Frischella*	Rhs_core _5<br>Rhs_core _9
Rhs_tox_023				1		*Gilliamella*	
Rhs_tox_024				1		*Gilliamella*	
Rhs_tox_025				2		*Gilliamella*	
Rhs_tox_026				8	*G. apis*		Rhs_core _4
Rhs_tox_027				14		*Snodgrassella*<br>*Gilliamella*	Rhs_core _5<br>Rhs_core _6
Rhs_tox_028	ParE_toxin superfamily	cl21503	0.001185	7	*Snodgrassella*	*Snodgrassella*<br>*Gilliamella*	Rhs_core _6
Rhs_tox_029	GIY-YIG_UPF0213	cd10456	0.007695	14		*Snodgrassella*<br>*Gilliamella*	
Rhs_tox_030				7		*Snodgrassella*<br>*Gilliamella*<br>*Apibacter*	
Rhs_tox_031				12		*Snodgrassella*	Rhs_core _6
Rhs_tox_032				2		*Snodgrassella*	
Rhs_tox_033				7		*Snodgrassella*	
Rhs_tox_034	Tox-ART-HYD1	pfam15633	4.09E-10	7	*Snodgrassella*	*Snodgrassella*	Rhs_core _6
Rhs_tox_035				1		*Snodgrassella*	

Table S1 continued

Toxin domain	CDD top hit	Accession	E-value	Copies	Genus (full length)	Genus (fragment)	Rhs core domains
Rhs_tox_036				2		*Snodgrassella Gilliamella*	Rhs_core _6
Rhs_tox_037	Colicin-DNase	pfam12639	7.25E-13	4		*Snodgrassella Gilliamella*	
Rhs_tox_038				27		*Snodgrassella Gilliamella*	
Rhs_tox_039	RHS_repeat	pfam05593	0.006908	1		*Snodgrassella*	Rhs_core _6
Rhs_tox_040				2	*Frischella*	*Frischella*	Rhs_core _5
Rhs_tox_041				19		*Snodgrassella Gilliamella*	Rhs_core _6
Rhs_tox_042				10		*Snodgrassella*	
Rhs_tox_043	GH-E	pfam14410	0.000944	17		*Snodgrassella Gilliamella Frischella*	
Rhs_tox_044	Ntox30	pfam15532	4.61E-05	25		*Snodgrassella Gilliamella*	
Rhs_tox_045				2	*Snodgrassella*	*Snodgrassella*	Rhs_core _6
Rhs_tox_046				19		*Snodgrassella Gilliamella*	Rhs_core _6
Rhs_tox_047	Colicin-DNase	pfam12639	4.11E-06	15	*Snodgrassella*	*Snodgrassella Gilliamella*	Rhs_core _6
Rhs_tox_048				9		*Snodgrassella*	Rhs_core _6
Rhs_tox_049				13		*Snodgrassella Gilliamella*	Rhs_core _6
Rhs_tox_050				17		*Snodgrassella*	Rhs_core _6
Rhs_tox_051				7	*Snodgrassella*	*Snodgrassella Gilliamella*	Rhs_core _6
Rhs_tox_052	HNHc	cd00085	0.0002	1		*Snodgrassella*	Rhs_core _6
Rhs_tox_053				1		*Snodgrassella*	Rhs_core _6
Rhs_tox_054				1		*Gilliamella*	
Rhs_tox_055	LHH	pfam14411	1.97E-36	5		*Snodgrassella*	
Rhs_tox_056				3		*Snodgrassella*	Rhs_core _6
Rhs_tox_057	Tox-HNH-EHHH	pfam15657	1.10E-06	13	*Snodgrassella*	*Snodgrassella Gilliamella*	Rhs_core _6
Rhs_tox_058				11	*Snodgrassella*	*Snodgrassella*	Rhs_core _6
Rhs_tox_059	Tox-ART-HYD1	pfam15633	2.35E-07	14		*Snodgrassella Gilliamella*	Rhs_core _6
Rhs_tox_060	DNase_NucA_NucB	pfam14040	1.21E-11	6		*Snodgrassella*	
Rhs_tox_061				21		*Snodgrassella Gilliamella*	Rhs_core _6
Rhs_tox_062				12		*Snodgrassella*	Rhs_core _6
Rhs_tox_063	Tox-GHH	pfam15636	1.36E-12	4		*Snodgrassella Gilliamella*	
Rhs_tox_064				7		*Snodgrassella*	
Rhs_tox_065	CdiA-CT_Ec-like	cd20692	3.31E-06	11		*Snodgrassella*	
Rhs_tox_066	ADPRTs_Tse2	pfam18648	6.61E-05	4		*Snodgrassella*	

Table S1 continued

Toxin domain	CDD top hit	Accession	E-value	Copies	Genus (full length)	Genus (fragment)	Rhs core domains
Rhs_tox_067				7	*Snodgrassella*	*Snodgrassella*	Rhs_core _6
Rhs_tox_068				2		*Snodgrassella*	
Rhs_tox_069				3		*Snodgrassella*	
Rhs_tox_070				9	*Snodgrassella*	*Snodgrassella*	Rhs_core _6
Rhs_tox_071				20	*Snodgrassella*	*Snodgrassella* *Gilliamella*	Rhs_core _6
Rhs_tox_072				26	*Snodgrassella*	*Snodgrassella* *Gilliamella*	Rhs_core _6
Rhs_tox_073				9	*Snodgrassella*	*Snodgrassella*	Rhs_core _6
Rhs_tox_074				12		*Snodgrassella* *Gilliamella*	Rhs_core _6
Rhs_tox_075				5		*Snodgrassella*	
Rhs_tox_076				4		*Snodgrassella*	
Rhs_tox_077	DUF4329	pfam14220	3.96E-13	10		*Snodgrassella* *Gilliamella*	Rhs_core _6
Rhs_tox_078	Tox-SHH	pfam15652	1.32E-35	7		*Snodgrassella* *Gilliamella*	
Rhs_tox_079	WHH	pfam14414	2.69E-12	4		*Snodgrassella*	
Rhs_tox_080				14		*Snodgrassella* *Gilliamella*	
Rhs_tox_081				5		*Snodgrassella*	
Rhs_tox_082				9		*Snodgrassella*	
Rhs_tox_083				2		*Snodgrassella*	
Rhs_tox_084				1		*Gilliamella*	
Rhs_tox_085	Ntox50	pfam15542	0.002308	2		*Snodgrassella*	
Rhs_tox_086				21		*Snodgrassella* *Gilliamella*	Rhs_core _10
Rhs_tox_087				3		*Snodgrassella*	Rhs_core _6
Rhs_tox_088				4	*Snodgrassella*	*Snodgrassella* *Gilliamella*	Rhs_core _6
Rhs_tox_089				4	*Snodgrassella*	*Snodgrassella*	Rhs_core _6
Rhs_tox_090				25		*Snodgrassella* *Gilliamella*	Rhs_core _6
Rhs_tox_091				7	*Snodgrassella*	*Snodgrassella*	Rhs_core _6
Rhs_tox_092				16	*Snodgrassella*	*Snodgrassella* *Gilliamella*	Rhs_core _6
Rhs_tox_093	OmpR	COG0745	0.005106	10	*Snodgrassella*	*Snodgrassella* *Gilliamella*	Rhs_core _6
Rhs_tox_094	Ntox8	pfam15545	9.39E-10	10	*Snodgrassella*	*Snodgrassella* *Gilliamella*	Rhs_core _6
Rhs_tox_095	Pput2613-deam	pfam14427	2.73E-54	2		*Snodgrassella*	
Rhs_tox_096	AHH	pfam14412	6.75E-06	3		*Gilliamella*	Rhs_core _6
Rhs_tox_097				22		*Snodgrassella* *Gilliamella*	Rhs_core _6
Rhs_tox_098				6		*Snodgrassella* *Gilliamella*	

Table S1 continued

Toxin domain	CDD top hit	Accession	E-value	Copies	Genus (full length)	Genus (fragment)	Rhs core domains
Rhs_tox_099	Toxin-deaminase	pfam14424	2.80E-18	5		*Snodgrassella*	
Rhs_tox_100	WHH	pfam14414	8.25E-15	4		*Snodgrassella*	Rhs_core _6
Rhs_tox_101	Colicin-DNase	pfam12639	1.81E-11	6	*Snodgrassella*	*Snodgrassella* *Gilliamella*	Rhs_core _6
Rhs_tox_102				23		*Snodgrassella* *Gilliamella*	
Rhs_tox_103	Tox-HNH-HHH	pfam15637	1.36E-05	3		*Snodgrassella*	
Rhs_tox_104	LHH	pfam14411	0.000668	4		*Snodgrassella* *Gilliamella*	
Rhs_tox_105				4		*Snodgrassella* *Gilliamella*	
Rhs_tox_106				14	*Snodgrassella*	*Snodgrassella*	Rhs_core _6
Rhs_tox_107				61		*Snodgrassella* *Gilliamella*	Rhs_core _6
Rhs_tox_108				3		*Snodgrassella*	
Rhs_tox_109	Tox-REase-3	pfam15647	2.01E-31	6		*Gilliamella*	
Rhs_tox_110	AHH	pfam14412	1.43E-16	17	*Snodgrassella*	*Snodgrassella* *Gilliamella*	Rhs_core _6
Rhs_tox_111				1		*Gilliamella*	
Rhs_tox_112				7	*Snodgrassella*	*Snodgrassella*	Rhs_core _6
Rhs_tox_113	AHH	pfam14412	1.31E-13	11	*Snodgrassella*	*Snodgrassella*	Rhs_core _6
Rhs_tox_114	CdiA-CT_Ec-like	cd20692	3.18E-35	2		*Gilliamella*	Rhs_core _4
Rhs_tox_115				3		*Snodgrassella*	
Rhs_tox_116				4		*Snodgrassella*	
Rhs_tox_117				1		*Gilliamella*	
Rhs_tox_118	CdiA-CT_Kp342-like	cd20724	2.27E-59	17	*Gilliamella*	*Snodgrassella* *Gilliamella*	Rhs_core _7
Rhs_tox_119	Colicin-DNase	pfam12639	2.45E-20	12	*Gilliamella*	*Gilliamella*	Rhs_core _7
Rhs_tox_120				3		*Gilliamella*	
Rhs_tox_121				1		*Gilliamella*	
Rhs_tox_122				4		*Gilliamella*	
Rhs_tox_123				1		*Gilliamella*	
Rhs_tox_124				5	*Frischella*	*Gilliamella*	Rhs_core _7
Rhs_tox_125	CdiA-CT_Ec-like	cd20686	9.56E-32	16		*Gilliamella* *Frischella*	
Rhs_tox_126				1		*Gilliamella*	
Rhs_tox_127				4		*Gilliamella*	
Rhs_tox_128				2		*Gilliamella*	
Rhs_tox_129	Peptidase_C39	pfam03412	2.28E-11	3	*Gilliamella*	*Gilliamella*	Rhs_core _7
Rhs_tox_130				8	*Gilliamella*	*Gilliamella*	Rhs_core _7
Rhs_tox_131				5		*Snodgrassella* *Gilliamella*	
Rhs_tox_132				8	*Gilliamella*	*Gilliamella*	Rhs_core _7
Rhs_tox_133				5		*Gilliamella*	

Table S1 continued

Toxin domain	CDD top hit	Accession	E-value	Copies	Genus (full length)	Genus (fragment)	Rhs core domains
Rhs_tox_134				3		*Gilliamella*	
Rhs_tox_135	EndoU_bacteria	pfam14436	7.53E-09	6		*Snodgrassella* *Gilliamella*	
Rhs_tox_136				1		*Gilliamella*	
Rhs_tox_137	DUF596	pfam04591	4.02E-13	18	*Gilliamella*	*Gilliamella* *Frischella*	Rhs_core _7
Rhs_tox_138				1		*Gilliamella*	
Rhs_tox_139	toxin-ParB	cd16392	7.81E-26	19	*Gilliamella*	*Gilliamella*	Rhs_core _7
Rhs_tox_140				6		*Gilliamella*	
Rhs_tox_141				1		*Gilliamella*	
Rhs_tox_142				3		*Gilliamella*	
Rhs_tox_143	CdiA-CT_Ec-like	cd20686	3.21E-51	17		*Snodgrassella* *Gilliamella*	
Rhs_tox_144	EndoU_bacteria	pfam14436	0.000303	13		*Snodgrassella* *Gilliamella*	
Rhs_tox_145				2		*Apibacter*	
Rhs_tox_146				4	*Gilliamella*	*Gilliamella*	Rhs_core _8
Rhs_tox_147				2	*Gilliamella* *Frischella*		Rhs_core _8
Rhs_tox_148				6	*Gilliamella*	*Gilliamella*	Rhs_core _8
Rhs_tox_149				6	*Snodgrassella*		Rhs_core _8
Rhs_tox_150				2		*Snodgrassella*	
Rhs_tox_151	Doc	COG3654	0.00062	6	*Snodgrassella*	*Snodgrassella*	Rhs_core _8
Rhs_tox_152				16	*Snodgrassella*	*Snodgrassella*	Rhs_core _8
Rhs_tox_153				3	*Snodgrassella*	*Snodgrassella*	Rhs_core _8
Rhs_tox_154				21	*Snodgrassella*	*Snodgrassella* *Gilliamella*	Rhs_core _8
Rhs_tox_155				2	*Snodgrassella*		Rhs_core _8
Rhs_tox_156				7	*Snodgrassella*	*Snodgrassella*	Rhs_core _8
Rhs_tox_157				2	*Snodgrassella*	*Snodgrassella*	Rhs_core _8
Rhs_tox_158				10		*Snodgrassella*	
Rhs_tox_159	MafB19-deam	pfam14437	6.23E-10	13	*Snodgrassella*	*Snodgrassella* *Schmidhempellia*	Rhs_core _8
Rhs_tox_160	Gln_amidase	pfam15644	3.04E-17	10	*Snodgrassella*	*Snodgrassella* *Gilliamella*	Rhs_core _8
Rhs_tox_161	LHH	pfam14411	1.09E-21	19	*Snodgrassella*	*Snodgrassella* *Gilliamella*	Rhs_core _8
Rhs_tox_162				5		*Apibacter* *Gilliamella*	
Rhs_tox_163				5		*Apibacter* *Gilliamella*	
Rhs_tox_164				27		*Snodgrassella* *Gilliamella* *Frischella*	Rhs_core _7 Rhs_core _9

Table S1 continued

Toxin domain	CDD top hit	Accession	E-value	Copies	Genus (full length)	Genus (fragment)	Rhs core domains
Rhs_tox_165	PBECR1	pfam18809	0.008097	11		*Snodgrassella*<br>*Gilliamella*<br>*Frischella*	
Rhs_tox_166				16		*Snodgrassella*<br>*Gilliamella*<br>*Frischella*	Rhs_core _7
Rhs_tox_167	AHH	pfam14412	1.32E-19	5		*Gilliamella*<br>*Frischella*	Rhs_core _9
Rhs_tox_168				2		*Gilliamella*	
Rhs_tox_169				6		*Gilliamella*	
Rhs_tox_170				7	*Gilliamella*	*Gilliamella*	Rhs_core _9
Rhs_tox_171	Tox-HNH-EHHH	pfam15657	1.99E-15	3		*Snodgrassella*<br>*Gilliamella*	
Rhs_tox_172				2		*Gilliamella*	Rhs_core _9
Rhs_tox_173				1		*Gilliamella*	Rhs_core _9
Rhs_tox_174	barnase	cd00933	3.15E-28	3	*Gilliamella*	*Gilliamella*	
Rhs_tox_175	Ntox8	pfam15545	3.34E-17	1		*Apibacter*	
Rhs_tox_176				31		*Snodgrassella*<br>*Gilliamella*	Rhs_core _6
Rhs_tox_177	Tox-HNH-EHHH	pfam15657	0.002963	10	*Apibacter*	*Snodgrassella*<br>*Gilliamella*	Rhs_core _10
Rhs_tox_178	AHH	pfam14412	0.000653	6		*Gilliamella*	
Rhs_tox_179				3		*Gilliamella*	
Rhs_tox_180	GIY-YIG_UvrC_Cho	cd10434	1.52E-05	1		*Gilliamella*	
Rhs_tox_181				3		*Gilliamella*	
Rhs_tox_182	AHH	pfam14412	1.58E-10	3		*Gilliamella*	
Rhs_tox_183	LHH	pfam14411	4.29E-17	3		*Gilliamella*	
Rhs_tox_184				6		*Gilliamella*	
Rhs_tox_185	WHH	pfam14414	2.70E-06	10	*Snodgrassella*	*Snodgrassella*<br>*Gilliamella*<br>*Apibacter*	
Rhs_tox_186	Tox-HNH-EHHH	pfam15657	2.53E-22	7	*Snodgrassella*	*Gilliamella*	Rhs_core _6<br>Rhs_core _10
Rhs_tox_187				3		*Gilliamella*	Rhs_core _10
Rhs_tox_188				21	*Snodgrassella*	*Snodgrassella*<br>*Gilliamella*	Rhs_core _6<br>Rhs_core _10
Rhs_tox_189				11	*Snodgrassella*	*Snodgrassella*	Rhs_core _8
Rhs_tox_190				6	*Snodgrassella*		Rhs_core _11
Rhs_tox_191				3		*Apibacter*	
Rhs_tox_192				1		*Apibacter*	
Rhs_tox_193	Lipase_3	cd00519	0.00084	1		*Apibacter*	
Rhs_tox_194				1		*Apibacter*	
Rhs_tox_195				1		*Apibacter*	
Rhs_tox_196				14	*Apibacter*	*Apibacter*	Rhs_core _12
Rhs_tox_197				8		*Apibacter*	

Table S1 continued

Toxin domain	CDD top hit	Accession	E-value	Copies	Genus (full length)	Genus (fragment)	Rhs core domains
Rhs_tox_198				3		*Apibacter*	
Rhs_tox_199				4		*Apibacter*	
Rhs_tox_200				4		*Apibacter*	
Rhs_tox_201				6		*Apibacter*	
Rhs_tox_202				8		*Apibacter*	Rhs_core _2
Rhs_tox_203				6	*Apibacter*	*Snodgrassella*	Rhs_core _2
Rhs_tox_204				1	*Apibacter*		Rhs_core _2
Rhs_tox_205				11	*Apibacter*	*Apibacter*	Rhs_core _2
Rhs_tox_206				4	*Apibacter*		Rhs_core _2
Rhs_tox_207				6		*Apibacter*	Rhs_core _2
Rhs_tox_208				6		*Apibacter*	Rhs_core _2
