# Supplementary material for: Evolution of Interbacterial Antagonism in Bee Gut Microbiota Reflects Host and Symbiont Diversification
Source: mSystems. 2021 May 11;6(3):e00063-21. doi: 10.1128/mSystems.00063-21 (PMC8125069; doi:10.1128/mSystems.00063-21)
Supplement: TABLE S2 [file mSystems.00063-21-st002.pdf]

Table S2. Proteins used in TssB, TssC, and TssH phylogenies.

Class	Species	T6SS subfamily	TssB	TssC	TssH
Alphaproteobacteria	*Rhizobacterium leguminosarum*	i3	WP_168301612.1	WP_130767081.1	WP_130698940.1
Alphaproteobacteria	*Agrobacterium tumefaciens*	i5	WP_042619947.1	WP_060724235.1	WP_137005461.1
Alphaproteobacteria	*Acidiphilium*	i5	WP_029312514.1	WP_029312513.1	WP_029312510.1
Alphaproteobacteria	*Acetobacter thailandicus*	i5	NHN93965.1	NHN93966.1	NHN93963.1
				NHN93967.1	
Alphaproteobacteria	*Roseomonas stagni*	i5	WP_092958817.1	WP_092958819.1	WP_092958814.1
				WP_092958821.1	
Alphaproteobacteria	*Inquilinus limosus*	i5	WP_034848252.1	WP_088155147.1	WP_088155175.1
			WP_088155151.1	WP_088155149.1	WP_088157277.1
				WP_088157287.1	
				WP_088157289.1	
Betaproteobacteria	*Snodgrassella alvi* wkB298	i3	WP_100141422.1	WP_100141421.1	WP_100152754.1
Betaproteobacteria	*Snodgrassella alvi* HK3	i3	WP_100099139.1	WP_100099140.1	WP_100123967.1
		i2	WP_100123960.1	WP_100123961.1	WP_100124084.1
Betaproteobacteria	*Snodgrassella alvi* wkB2	i3	WP_025329757.1	WP_025329758.1	WP_025329756.1
		i2	WP_025330951.1	WP_038648915.1	WP_025330944.1
Betaproteobacteria	*Burkholderia thailandensis* E264	i1, i3, i4b	WP_009888592.1	WP_009888594.1	WP_009893771.1
			WP_009894728.1	WP_009894729.1	WP_009895032.1
			WP_009895017.1	WP_009895019.1	WP_009896189.1
			WP_009896198.1	WP_043037857.1	WP_009907099.1
			WP_009901015.1	WP_009901014.1	WP_009910486.1
Betaproteobacteria	*Neisseria mucosa*	i2	WP_003760099.1	WP_060975453.1	WP_060975457.1
Betaproteobacteria	*Amantichitinum ursilacus*	i2, i4a	WP_053938100.1	WP_053938099.1	WP_053938104.1
			WP_053939065.1	WP_053939066.1	WP_053939056.1
Betaproteobacteria	*Cupriavidus basilensis*	i2, i3	WP_043344071.1	WP_043344074.1	WP_043344068.1
			WP_043353035.1	WP_043353033.1	WP_043353022.1
Betaproteobacteria	Burkholderia agricolaris	i1, i2, i4b	WP_153135049.1	WP_153135050.1	WP_153135055.1
			WP_153139628.1	WP_153139630.1	WP_153140969.1
			WP_153140974.1	WP_153140973.1	
Betaproteobacteria	Burkholderia hayleyella	i1, i3	WP_153101208.1	WP_153101210.1	WP_153101206.1
			WP_153102337.1	WP_153102336.1	WP_153102338.1
Betaproteobacteria	Burkholderia bonniea	i1, i2	WP_153074100.1	WP_153074099.1	
			WP_153075580.1	WP_153075579.1	
Betaproteobacteria	Bordetella avium	i4a	WP_119647618.1	WP_119647619.1	WP_119536710.1
Betaproteobacteria	Cupriavidus taiwanensis	i3	WP_115679530.1	WP_115679531.1	WP_115679529.1
Deltaproteobacteria	Geobacter sp. M21	i4a	WP_015836522.1	WP_015836523.1	WP_012774279.1
Epsilonproteobacteria	Campylobacter jejuni	i1	WP_087700196.1	WP_126265123.1	
Gammaproteobacteria	*Salmonella enterica* subsp. enterica serovar Typhimurium LT2	i3	NP_459270.1	NP_459271.1	NP_459269.1
Gammaproteobacteria	*Enterobacter cloacae*	**i3**	WP_047024474.1	WP_063861990.1	WP_063866598.1
Gammaproteobacteria	*Cronobacter sakazakii*	i2, i3	WP_007849582.1	WP_004385964.1	WP_007889015.1
			WP_007853510.1	WP_085958991.1	
Gammaproteobacteria	*Pantoea alhagi* LTYR-11Z	i3	WP_085068116.1	WP_085068115.1	WP_085068100.1
			WP_085069784.1	WP_085069786.1	WP_085069774.1
Gammaproteobacteria	*Serratia marcescens* kz11	i3	WP_015378282.1	WP_004934958.1	WP_103687201.1
			WP_004935376.1	WP_004935371.1	WP_103687226.1
Gammaproteobacteria	*Pseudomonas aeruginosa* PAO1	i1, i3, i4a	NP_248773.1	NP_250349.1	NP_248780.1
			NP_251055.1	NP_248774.1	NP_250353.1
					NP_251061.1
Gammaproteobacteria	*Gilliamella apicola* wkB1	i2	WP_025316042.1	WP_025316041.1	WP_025316036.1
Gammaproteobacteria	*Gilliamella* sp. A7	i2	WP_086363296.1	WP_086363295.1	WP_086363290.1
Gammaproteobacteria	*Gilliamella* sp. wkB308	i2	WP_065559521.1	WP_065559520.1	WP_065559515.1
Gammaproteobacteria	*Frischella perrara* PEB0191	i1, i2	WP_039105357.1	WP_039105358.1	WP_039105368.1
			WP_039105671.1	WP_039105670.1	WP_082018306.1
Gammaproteobacteria	*Candidatus* "Schmidhempelia bombi"	i2	WP_024496317.1	WP_024496316.1	WP_024496311.1
Gammaproteobacteria	*Pragia fontium*	i1, i2	WP_047780369.1	WP_047780368.1	WP_047780362.1
			WP_047780835.1	WP_047780836.1	WP_082118954.1
Gammaproteobacteria	*Pseudoalteromonas luteoviolacea*	i2	WP_039610623.1	WP_039610624.1	WP_046357733.1
Gammaproteobacteria	*Escherichia coli* O157:H7 EDL933	i1	WP_000037399.1	WP_000056994.1	WP_000614374.1

Table S2 continued

Class	Species	T6SS subfamily	TssB	TssC	TssH
Gammaproteobacteria	*Aliivibrio fischeri* FQ-A001	i1	WP_005418723.1	WP_011261636.1	WP_133415340.1
			WP_012535409.1	WP_063645634.1	WP_133416029.1
Gammaproteobacteria	*Vibrio cholerae* 10432-62	i1	WP_000031391.1	WP_032469832.1	WP_032469833.1
Gammaproteobacteria	*Aeromonas hydrophila*	i1	WP_130632564.1	WP_139389892.1	WP_168234847.1
Gammaproteobacteria	*Dickeya dadantii* DSM 18020	i1	WP_013317138.1	WP_013317139.1	WP_038910671.1
Gammaproteobacteria	*Shewanella psychrophila* WP2	i1	WP_077751561.1	WP_077751560.1	WP_077751552.1
Gammaproteobacteria	*Acinetobacter baumannii* ATCC 19606	i4b	WP_001119042.1	WP_001066523.1	WP_100223335.1
Gammaproteobacteria	*Pseudomonas putida* 1290	i1, i3	WP_050703367.1	WP_136913317.1	WP_136913314.1
			WP_136914036.1	WP_136914035.1	
Gammaproteobacteria	*Pseudomonas putida* KT2440	i4a	AAN68708.1	AAN68231.1	AAN68703.1
Gammaproteobacteria	*Edwardsiella anguillarum*	i2, i4a	WP_034166143.1	WP_034162666.1	WP_081926387.1
			WP_045424188.1	WP_038631670.1	WP_051905104.1
Gammaproteobacteria	*Acinetobacter baylyi*	i4b	WP_004929014.1	WP_120430446.1	WP_120430447.1
Gammaproteobacteria	*Salmonella bongori*	i1, i4a	HAB1658684.1	HAB1658685.1	HAB1658690.1
			HAB1661735.1	HAB1661734.1	HAB1661726.1
Gammaproteobacteria	*Gilliamella* sp. Choc5-1	i1	OCG49108.1	OCG49107.1	OCG49100.1
Gammaproteobacteria	*Gilliamella* sp. ESL0182	i1	WP_110432959.1	WP_110432960.1	WP_110432968.1
Gammaproteobacteria	*Frischella perrara* ESL0167	i1	WP_110444015.1	WP_110444016.1	WP_110444024.1
Flavobacteriia	*Apibacter adventoris* wkB180	iii	WP_105193632.1	WP_105193635.1	WP_105193633.1
Flavobacteriia	*Apibacter muscae*	iii	WP_146262280.1	WP_146262279.1	WP_146310390.1
Flavobacteriia	*Apibacter mensalis*	iii	WP_055426258.1	WP_141656254.1	WP_055426257.1
Flavobacteriia	*Flavobacterium johnsoniae* DSM 2064	iii	ABQ06283.1	ABQ06282.1	ABQ06297.1
Flavobacteriia	*Chryseobacterium aquaticum*	iii	WP_082171026.1	WP_056017893.1	WP_056017858.1
			WP_056017271.1	WP_056017274.1	WP_056016891.1
Bacteroidia	*Bacteroides fragilis* 638R	iii	CBW22515.1	CBW22514.1	CBW22513.1
Bacteroidia	*Bacteroides caccae* CL03T12C61	iii	EIY16150.1	EIY16149.1	EIY16154.1
Bacteroidia	*Bacteroides uniformis* CL03T00C23	iii	EIY72195.1	EIY72196.1	EIY72192.1
