# Supplementary material for: Evolution of Interbacterial Antagonism in Bee Gut Microbiota Reflects Host and Symbiont Diversification
Source: mSystems. 2021 May 11;6(3):e00063-21. doi: 10.1128/mSystems.00063-21 (PMC8125069; doi:10.1128/mSystems.00063-21)
Supplement: TABLE S3 [file mSystems.00063-21-st003.pdf]

**Table S3.** Bee gut isolate genomes analyzed in this study.

| Assembly accession | Strain | Species | Host species | Location |
|---|---|---|---|---|
| GCF_002964965.1 | wkB180 | *Apibacter adventoris* | *Apis dorsata* | Southeast Asia |
| GCF_002964975.1 | wkB301 | *Apibacter adventoris* | *Apis dorsata* | Southeast Asia |
| GCF_001418685.1 | R-53146 | *Apibacter mensalis* | *Bombus lapidarius* | Europe |
| GCF_009827835.1 | B2912 | *Apibacter* sp. | *Apis cerana* | China |
| GCF_009828155.1 | B3239 | *Apibacter* sp. | *Apis cerana* | China |
| GCF_009827885.1 | B3546 | *Apibacter* sp. | *Apis cerana* | China |
| GCF_009827845.1 | B3813 | *Apibacter* sp. | *Apis cerana* | China |
| GCF_009827855.1 | B3883 | *Apibacter* sp. | *Apis cerana* | China |
| GCF_009828165.1 | B3887 | *Apibacter* sp. | *Apis cerana* | China |
| GCF_009827795.1 | B3889 | *Apibacter* sp. | *Apis cerana* | China |
| GCF_009827805.1 | B3912 | *Apibacter* sp. | *Apis cerana* | China |
| GCF_009827735.1 | B3913 | *Apibacter* sp. | *Apis cerana* | China |
| GCF_009827755.1 | B3918 | *Apibacter* sp. | *Apis cerana* | China |
| GCF_009827745.1 | B3924 | *Apibacter* sp. | *Apis cerana* | China |
| GCF_009827705.1 | B3935 | *Apibacter* sp. | *Apis cerana* | China |
| GCF_002964915.1 | wkB309 | *Apibacter* sp. | *Apis cerana* | Southeast Asia |
| GCF_002007565.1 | BBC0122 | *Bartonella apis* | *Apis mellifera* | Europe |
| GCF_002007485.1 | BBC0178 | *Bartonella apis* | *Apis mellifera* | Europe |
| GCF_002007505.1 | BBC0244 | *Bartonella apis* | *Apis mellifera* | Europe |
| GCF_001952075.1 | PEB0122 | *Bartonella apis* | *Apis mellifera* | North America |
| GCF_001952065.1 | PEB0149 | *Bartonella apis* | *Apis mellifera* | North America |
| GCF_001952045.1 | PEB0150 | *Bartonella apis* | *Apis mellifera* | North America |
| GCF_000471645.3 | Bimp | *Candidatus* Schmidhempelia bombi | *Bombus impatiens* | North America |
| GCF_003202705.1 | ESL0167 | *Frischella perrara* | *Apis mellifera* | Europe |
| GCF_000807275.1 | PEB0191 | *Frischella perrara* | *Apis mellifera* | North America |
| GCF_002141515.1 | A-1-24 | *Gilliamella apicola* | *Apis mellifera* | North America |
| GCF_002141675.1 | A-12-12 | *Gilliamella apicola* | *Apis mellifera* | North America |
| GCF_002141565.1 | A-2-24 | *Gilliamella apicola* | *Apis mellifera* | North America |
| GCF_002141655.1 | A-7-12 | *Gilliamella apicola* | *Apis mellifera* | North America |
| GCF_002141665.1 | A-7-24 | *Gilliamella apicola* | *Apis mellifera* | North America |
| GCF_002141715.1 | A-8-12 | *Gilliamella apicola* | *Apis mellifera* | North America |
| GCF_002141745.1 | A-9-12 | *Gilliamella apicola* | *Apis mellifera* | North America |
| GCF_002141555.1 | A8 | *Gilliamella apicola* | *Apis mellifera* | North America |
| GCF_002141735.1 | A9 | *Gilliamella apicola* | *Apis mellifera* | North America |
| GCF_002141905.1 | AM4 | *Gilliamella apicola* | *Apis mellifera* | North America |
| GCF_002141575.1 | AM6 | *Gilliamella apicola* | *Apis mellifera* | North America |
| GCF_002141595.1 | Aw-17 | *Gilliamella apicola* | *Apis mellifera* | North America |
| GCF_002141815.1 | AW11 | *Gilliamella apicola* | *Apis mellifera* | North America |
| GCF_002141755.1 | AW13 | *Gilliamella apicola* | *Apis mellifera* | North America |
| GCF_003202815.1 | ESL0178 | *Gilliamella apicola* | *Apis mellifera* | Europe |
| GCF_002088825.1 | N-12-12 | *Gilliamella apicola* | *Apis mellifera* | Europe |
| GCF_002088905.1 | N-15-12 | *Gilliamella apicola* | *Apis mellifera* | Europe |
| GCF_002142285.1 | N-22 | *Gilliamella apicola* | *Apis mellifera* | Europe |
| GCF_002142345.1 | N-28 | *Gilliamella apicola* | *Apis mellifera* | Europe |
| GCF_002088895.1 | N-9-4 | *Gilliamella apicola* | *Apis mellifera* | Europe |
| GCF_002142265.1 | N-G5 | *Gilliamella apicola* | *Apis mellifera* | Europe |
| GCF_002141785.1 | N10 | *Gilliamella apicola* | *Apis mellifera* | Europe |
| GCF_002141855.1 | N2 | *Gilliamella apicola* | *Apis mellifera* | Europe |
| GCF_002141635.1 | N4 | *Gilliamella apicola* | *Apis mellifera* | Europe |
| GCF_002141885.1 | N6 | *Gilliamella apicola* | *Apis mellifera* | Europe |
| GCF_002088845.1 | NO10 | *Gilliamella apicola* | *Apis mellifera* | Europe |
| GCF_002088855.1 | NO5 | *Gilliamella apicola* | *Apis mellifera* | Europe |
| GCF_002088795.1 | NO6 | *Gilliamella apicola* | *Apis mellifera* | Europe |
| GCF_002088815.1 | NO8 | *Gilliamella apicola* | *Apis mellifera* | Europe |
| GCF_001690735.1 | PEB0154 | *Gilliamella apicola* | *Apis mellifera* | North America |
| GCF_007559145.1 | W8127 | *Gilliamella apicola* | *Apis mellifera* | China |
| GCF_007559165.1 | W8131 | *Gilliamella apicola* | *Apis mellifera* | China |
| GCF_000599985.1 | wkB1 | *Gilliamella apicola* | *Apis mellifera* | North America |
| GCF_001693435.1 | wkB7 | *Gilliamella apicola* | *Apis mellifera* | North America |
| GCF_002141865.1 | A-4-12 | *Gilliamella apis* | *Apis mellifera* | North America |
| GCF_002142035.1 | A-TSA1 | *Gilliamella apis* | *Apis mellifera* | North America |
| GCF_002142055.1 | A-TSA2 | *Gilliamella apis* | *Apis mellifera* | North America |
| GCF_002142065.1 | A-TSA3 | *Gilliamella apis* | *Apis mellifera* | North America |
| GCF_002142135.1 | A-TSA4 | *Gilliamella apis* | *Apis mellifera* | North America |
| GCF_002141825.1 | AM1 | *Gilliamella apis* | *Apis mellifera* | North America |

**Table S3, continued**

| Assembly accession | Strain | Species | Host species | Location |
|---|---|---|---|---|
| GCF_003202875.1 | ESL0169 | *Gilliamella apis* | *Apis mellifera* | Europe |
| GCF_001690775.1 | M1-2G | *Gilliamella apis* | *Apis mellifera* | North America |
| GCF_002142185.1 | N-G1 | *Gilliamella apis* | *Apis mellifera* | Europe |
| GCF_002142225.1 | N-G3 | *Gilliamella apis* | *Apis mellifera* | Europe |
| GCF_002142255.1 | N-G4 | *Gilliamella apis* | *Apis mellifera* | Europe |
| GCF_002141945.1 | NO1 | *Gilliamella apis* | *Apis mellifera* | Europe |
| GCF_002142085.1 | NO12 | *Gilliamella apis* | *Apis mellifera* | Europe |
| GCF_002141975.1 | NO13 | *Gilliamella apis* | *Apis mellifera* | Europe |
| GCF_002142115.1 | NO14 | *Gilliamella apis* | *Apis mellifera* | Europe |
| GCF_002141935.1 | NO15 | *Gilliamella apis* | *Apis mellifera* | Europe |
| GCF_002141985.1 | NO16 | *Gilliamella apis* | *Apis mellifera* | Europe |
| GCF_002142155.1 | NO3 | *Gilliamella apis* | *Apis mellifera* | Europe |
| GCF_002142165.1 | NO4 | *Gilliamella apis* | *Apis mellifera* | Europe |
| GCF_001690755.1 | PEB0162 | *Gilliamella apis* | *Apis mellifera* | North America |
| GCF_001690175.1 | PEB0183 | *Gilliamella apis* | *Apis mellifera* | North America |
| GCF_900103255.1 | G. bombi | *Gilliamella bombi* | *Bombus* sp. | Europe |
| GCF_900094945.1 | R-53248 | *Gilliamella bombicola* | *Bombus* sp. | Europe |
| GCF_900094935.1 | R-53144 | *Gilliamella intestini* | *Bombus* sp. | Europe |
| GCF_900103085.1 | G. mensalis | *Gilliamella mensalis* | *Bombus* sp. | Europe |
| GCF_002142015.1 | A7 | *Gilliamella* sp. | *Apis mellifera* | North America |
| GCF_001690255.1 | App2-1 | *Gilliamella* sp. | *Bombus appositus* | North America |
| GCF_001690495.1 | App4-10 | *Gilliamella* sp. | *Bombus appositus* | North America |
| GCF_001690525.1 | App6-5 | *Gilliamella* sp. | *Bombus appositus* | North America |
| GCF_001690535.1 | Bif1-4 | *Gilliamella* sp. | *Bombus bifarius* | North America |
| GCF_001690835.1 | Bim1-2 | *Gilliamella* sp. | *Bombus bimaculatus* | North America |
| GCF_001690575.1 | Bim3-2 | *Gilliamella* sp. | *Bombus bimaculatus* | North America |
| GCF_001690585.1 | Choc3-5 | *Gilliamella* sp. | *Bombus impatiens* | North America |
| GCF_001690355.1 | Choc4-2 | *Gilliamella* sp. | *Bombus impatiens* | North America |
| GCF_001690595.1 | Choc5-1 | *Gilliamella* sp. | *Bombus impatiens* | North America |
| GCF_001690385.1 | Choc6-1 | *Gilliamella* sp. | *Bombus impatiens* | North America |
| GCF_003202775.1 | ESL0172 | *Gilliamella* sp. | *Apis mellifera* | Europe |
| GCF_003202915.1 | ESL0177 | *Gilliamella* sp. | *Apis mellifera* | Europe |
| GCF_003202655.1 | ESL0182 | *Gilliamella* sp. | *Apis mellifera* | Europe |
| GCF_001690825.1 | Fer1-1 | *Gilliamella* sp. | *Bombus fervidus* | North America |
| GCF_001690305.1 | Fer2-1 | *Gilliamella* sp. | *Bombus fervidus* | North America |
| GCF_001690515.1 | Fer4-1 | *Gilliamella* sp. | *Bombus fervidus* | North America |
| GCF_001690815.1 | GillExp13 | *Gilliamella* sp. | *Bombus impatiens* | North America |
| GCF_001690335.1 | Gris1-4 | *Gilliamella* sp. | *Bombus griseocollis* | North America |
| GCF_001690345.1 | Gris3-2 | *Gilliamella* sp. | *Bombus griseocollis* | North America |
| GCF_001690265.1 | HK2 | *Gilliamella* sp. | *Bombus pensylvanicus* | North America |
| GCF_001690795.1 | HK7 | *Gilliamella* sp. | *Bombus pensylvanicus* | North America |
| GCF_001690415.1 | Imp1-1 | *Gilliamella* sp. | *Bombus impatiens* | North America |
| GCF_001690425.1 | Imp1-6 | *Gilliamella* sp. | *Bombus impatiens* | North America |
| GCF_009795895.1 | Lep-s21 | *Gilliamella* sp. | *Bombus lepidus* | China |
| GCF_009795845.1 | Lep-s35 | *Gilliamella* sp. | *Bombus lepidus* | China |
| GCF_009795905.1 | Lep-s5 | *Gilliamella* sp. | *Bombus lepidus* | China |
| GCF_002142215.1 | N-G2 | *Gilliamella* sp. | *Apis mellifera* | Europe |
| GCF_002142275.1 | N-W3 | *Gilliamella* sp. | *Apis mellifera* | Europe |
| GCF_001690435.1 | Nev3-1 | *Gilliamella* sp. | *Bombus nevadensis* | North America |
| GCF_001690895.1 | Nev5-1 | *Gilliamella* sp. | *Bombus nevadensis* | North America |
| GCF_001690605.1 | Nev6-6 | *Gilliamella* sp. | *Bombus nevadensis* | North America |
| GCF_001690655.1 | Occ3-1 | *Gilliamella* sp. | *Bombus occientalis* | North America |
| GCF_001690675.1 | Occ4-3 | *Gilliamella* sp. | *Bombus occientalis* | North America |
| GCF_009795885.1 | Pas-s25 | *Gilliamella* sp. | *Bombus pascuorum* | China |
| GCF_009795865.1 | Pas-s27 | *Gilliamella* sp. | *Bombus pascuorum* | China |
| GCF_009795795.1 | Pas-s95 | *Gilliamella* sp. | *Bombus pascuorum* | China |
| GCF_009795805.1 | Pra-s52 | *Gilliamella* sp. | *Bombus pratorum* | China |
| GCF_009795785.1 | Pra-s54 | *Gilliamella* sp. | *Bombus pratorum* | China |
| GCF_009795755.1 | Pra-s60 | *Gilliamella* sp. | *Bombus pratorum* | China |
| GCF_009795745.1 | Pra-s65 | *Gilliamella* sp. | *Bombus pratorum* | China |
| GCF_001690275.1 | WF3-4 | *Gilliamella* sp. | *Bombus pensylvanicus* | North America |
| GCF_001690195.1 | wkB108 | *Gilliamella* sp. | *Apis dorsata* | Southeast Asia |
| GCF_000733115.1 | wkB11 | *Gilliamella* sp. | *Bombus bimaculatus* | North America |
| GCF_001690685.1 | wkB112 | *Gilliamella* sp. | *Apis dorsata* | Southeast Asia |
| GCF_001693755.1 | wkB171 | *Gilliamella* sp. | *Apis adreniformis* | Southeast Asia |
| GCF_001690705.1 | wkB178 | *Gilliamella* sp. | *Apis dorsata* | Southeast Asia |
| GCF_001690875.1 | wkB18 | *Gilliamella* sp. | *Bombus vagans* | North America |
| GCF_001690445.1 | wkB195 | *Gilliamella* sp. | *Apis cerana* | Southeast Asia |

**Table S3, continued**

| Assembly accession | Strain | Species | Host species | Location |
|---|---|---|---|---|
| GCF_001690235.1 | wkB292 | *Gilliamella* sp. | *Apis cerana* | Southeast Asia |
| GCF_000695585.1 | wkB30 | *Gilliamella* sp. | *Bombus vagans* | North America |
| GCF_001690185.1 | wkB308 | *Gilliamella* sp. | *Apis cerana* | Southeast Asia |
| GCF_001693745.1 | wkB72 | *Gilliamella* sp. | *Apis cerana* | Southeast Asia |
| GCF_002917995.1 | A29 | *Parasaccharibacter apium* | *Apis mellifera* (larvae) | North America |
| GCF_002592045.1 | AS1 | *Parasaccharibacter apium* | *Apis mellifera* (larvae) | North America |
| GCF_002917945.1 | B8 | *Parasaccharibacter apium* | *Apis mellifera* (larvae) | North America |
| GCF_002917985.1 | C6 | *Parasaccharibacter apium* | *Apis mellifera* (larvae) | North America |
| GCF_002079945.1 | G7_7_3c | *Parasaccharibacter apium* | *Apis mellifera* (larvae) | North America |
| GCF_002088735.1 | A-1-12 | *Snodgrassella alvi* | *Apis mellifera* | North America |
| GCF_002088405.1 | A-10-12 | *Snodgrassella alvi* | *Apis mellifera* | North America |
| GCF_002088695.1 | A-11-12 | *Snodgrassella alvi* | *Apis mellifera* | North America |
| GCF_002088585.1 | A-2-12 | *Snodgrassella alvi* | *Apis mellifera* | North America |
| GCF_002088465.1 | A-5-24 | *Snodgrassella alvi* | *Apis mellifera* | North America |
| GCF_002088515.1 | A-9-24 | *Snodgrassella alvi* | *Apis mellifera* | North America |
| GCF_002089015.1 | A11 | *Snodgrassella alvi* | *Apis mellifera* | North America |
| GCF_002088675.1 | A12 | *Snodgrassella alvi* | *Apis mellifera* | North America |
| GCF_002088395.1 | A2 | *Snodgrassella alvi* | *Apis mellifera* | North America |
| GCF_002088455.1 | A3 | *Snodgrassella alvi* | *Apis mellifera* | North America |
| GCF_002088475.1 | A5 | *Snodgrassella alvi* | *Apis mellifera* | North America |
| GCF_002777315.1 | App2-2 | *Snodgrassella alvi* | *Bombus appositus* | North America |
| GCF_002777425.1 | App4-8 | *Snodgrassella alvi* | *Bombus appositus* | North America |
| GCF_002777465.1 | App6-4 | *Snodgrassella alvi* | *Bombus appositus* | North America |
| GCF_002088415.1 | Aw-18 | *Snodgrassella alvi* | *Apis mellifera* | North America |
| GCF_002019415.1 | Aw-20 | *Snodgrassella alvi* | *Apis mellifera* | North America |
| GCF_002406645.1 | E1 | *Snodgrassella alvi* | *Apis mellifera* | North America |
| GCF_003202885.1 | ESL0196 | *Snodgrassella alvi* | *Apis mellifera* | Europe |
| GCF_002777415.1 | Fer1-2 | *Snodgrassella alvi* | *Bombus fervidus* | North America |
| GCF_002777485.1 | Fer2-2 | *Snodgrassella alvi* | *Bombus fervidus* | North America |
| GCF_002777495.1 | Fer4-2 | *Snodgrassella alvi* | *Bombus fervidus* | North America |
| GCF_002777525.1 | Gris1-3 | *Snodgrassella alvi* | *Bombus griseocollis* | North America |
| GCF_002777615.1 | Gris1-6 | *Snodgrassella alvi* | *Bombus griseocollis* | North America |
| GCF_002777335.1 | Gris2-3-4 | *Snodgrassella alvi* | *Bombus griseocollis* | North America |
| GCF_002777595.1 | Gris3-4 | *Snodgrassella alvi* | *Bombus griseocollis* | North America |
| GCF_002777705.1 | HK3 | *Snodgrassella alvi* | *Bombus pensylvanicus* | North America |
| GCF_002777795.1 | HK9x | *Snodgrassella alvi* | *Bombus pensylvanicus* | North America |
| GCF_002777925.1 | MS1-3 | *Snodgrassella alvi* | *Apis mellifera* | North America |
| GCF_002088655.1 | N-23 | *Snodgrassella alvi* | *Apis mellifera* | Europe |
| GCF_002088755.1 | N-S1 | *Snodgrassella alvi* | *Apis mellifera* | Europe |
| GCF_002088595.1 | N-S2 | *Snodgrassella alvi* | *Apis mellifera* | Europe |
| GCF_002088635.1 | N-S3 | *Snodgrassella alvi* | *Apis mellifera* | Europe |
| GCF_002088665.1 | N-S4 | *Snodgrassella alvi* | *Apis mellifera* | Europe |
| GCF_002088765.1 | N-S5 | *Snodgrassella alvi* | *Apis mellifera* | Europe |
| GCF_002088525.1 | N-W4 | *Snodgrassella alvi* | *Apis mellifera* | Europe |
| GCF_002088555.1 | N-W7 | *Snodgrassella alvi* | *Apis mellifera* | Europe |
| GCF_002088575.1 | N9 | *Snodgrassella alvi* | *Apis mellifera* | Europe |
| GCF_002777575.1 | Nev3CBA3 | *Snodgrassella alvi* | *Bombus nevadensis* | North America |
| GCF_002777825.1 | Nev4-2 | *Snodgrassella alvi* | *Bombus nevadensis* | North America |
| GCF_002777635.1 | Occ4-2 | *Snodgrassella alvi* | *Bombus occientalis* | North America |
| GCF_002777865.1 | PEB0171 | *Snodgrassella alvi* | *Apis mellifera* | North America |
| GCF_002777875.1 | PEB0178 | *Snodgrassella alvi* | *Apis mellifera* | North America |
| GCF_002777325.1 | Pens2-2-5 | *Snodgrassella alvi* | *Bombus pensylvanicus* | North America |
| GCF_900094895.1 | R-53583 | *Snodgrassella alvi* | *Bombus* sp. | Europe |
| GCF_002777675.1 | Ruf1-X | *Snodgrassella alvi* | *Bombus rufocinctus* | North America |
| GCF_002777345.1 | Snod2-1-5 | *Snodgrassella alvi* | *Bombus impatiens* | North America |
| GCF_002777745.1 | WF3-3 | *Snodgrassella alvi* | *Bombus pensylvanicus* | North America |
| GCF_000695565.1 | wkB12 | *Snodgrassella alvi* | *Bombus bimaculatus* | North America |
| GCF_000600005.1 | wkB2 | *Snodgrassella alvi* | *Apis mellifera* | North America |
| GCF_002777775.1 | wkB237 | *Snodgrassella alvi* | *Apis andreniformis* | Southeast Asia |
| GCF_002777655.1 | wkB273 | *Snodgrassella alvi* | *Apis florea* | Southeast Asia |
| GCF_000695545.1 | wkB29 | *Snodgrassella alvi* | *Bombus vagans* | North America |
| GCF_002777855.1 | wkB298 | *Snodgrassella alvi* | *Apis cerana* | Southeast Asia |
| GCF_002777695.1 | wkB332 | *Snodgrassella alvi* | *Apis mellifera* | Southeast Asia |
| GCF_002777815.1 | wkB339 | *Snodgrassella alvi* | *Apis mellifera* | Southeast Asia |
| GCF_002777735.1 | wkB9 | *Snodgrassella alvi* | *Apis mellifera* | North America |
